# Arachidonic acid, a clinically adverse mediator in the ovarian cancer microenvironment, impairs JAK‐STAT signaling in macrophages by perturbing lipid raft structures

**DOI:** 10.1002/1878-0261.13221

**Published:** 2022-05-04

**Authors:** Mohamad K. Hammoud, Raimund Dietze, Jelena Pesek, Florian Finkernagel, Annika Unger, Tim Bieringer, Andrea Nist, Thorsten Stiewe, Aditya M. Bhagwat, Wolfgang Andreas Nockher, Silke Reinartz, Sabine Müller‐Brüsselbach, Johannes Graumann, Rolf Müller

**Affiliations:** ^1^ Center for Tumor Biology and Immunology Philipps University Marburg Germany; ^2^ Medical Mass Spectrometry Core Facility Philipps University Marburg Germany; ^3^ Genomics Core Facility Philipps University Marburg Germany; ^4^ Max‐Planck‐Institute for Heart and Lung Research Bad Nauheim Germany; ^5^ The German Centre for Cardiovascular Research (DZHK), Partner Site Rhine‐Main Max Planck Institute for Heart and Lung Research Bad Nauheim Germany; ^6^ Present address: Hochschule Landshut Landshut 84036 Germany; ^7^ Present address: Institute for Translational Proteomics Philipps University Marburg Germany

**Keywords:** arachidonic acid, interferon, lipid rafts, macrophage, ovarian cancer microenvironment, STAT

## Abstract

Survival of ovarian carcinoma is associated with the abundance of immunosuppressed CD163^high^CD206^high^ tumor‐associated macrophages (TAMs) and high levels of arachidonic acid (AA) in the tumor microenvironment. Here, we show that both associations are functionally linked. Transcriptional profiling revealed that high *CD163* and *CD206/MRC1* expression in TAMs is strongly associated with an inhibition of cytokine‐triggered signaling, mirrored by an impaired transcriptional response to interferons and IL‐6 in monocyte‐derived macrophages by AA. This inhibition of pro‐inflammatory signaling is caused by dysfunctions of the cognate receptors, indicated by the inhibition of JAK1, JAK2, STAT1, and STAT3 phosphorylation, and by the displacement of the interferon receptor IFNAR1, STAT1 and other immune‐regulatory proteins from lipid rafts. AA exposure led to a dramatic accumulation of free AA in lipid rafts, which appears to be mechanistically crucial, as the inhibition of its incorporation into phospholipids did not affect the AA‐mediated interference with STAT1 phosphorylation. Inhibition of interferon‐triggered STAT1 phosphorylation by AA was reversed by water‐soluble cholesterol, known to prevent the perturbation of lipid raft structure by AA. These findings suggest that the pharmacologic restoration of lipid raft functions in TAMs may contribute to the development new therapeutic approaches.

## Graphical abstract

This study shows that the clinically adverse abundance of immunocompromised macrophages and arachidonic acid (AA) in the ovarian carcinoma microenvironment (TME) are functionally linked. AA impairs transcriptional signalling via JAK/STAT‐dependent cytokine receptors by counteracting compartmentalization of receptor and STAT proteins into lipid rafts. Inhibition of STAT signalling is reversible by protecting lipid raft structure with water‐soluble cholesterol, pointing to potential therapeutic applications.

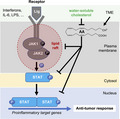

**Abbreviations**
AA: arachidonic acid
Chol/MCD: cholesterol/methyl‐β‐cyclodextrin complex
CPM: counts per million
DAPI: 4′,6‐diamidin‐2‐phenylindol
ETYA: inhibitor 5,8,11,14‐eicosatetraynoic acid
FC: fold cahge
HGSC: high‐grade serous carcinoma: inversion
IFN: interferon
LPS: lipopolysaccharide
MDM: monocyte‐derived macrophage
NGS: next‐generation sequencing
OC: ovarian cancer
OS: overall survival
PUFA: polyunsaturated fatty acid
RFS: relapse‐free survival
RNA‐Seq: RNA‐sequencing
RT‐qPCR: quantitative reverse transcriptase PCR
TAM: tumor‐associated macrophage

## 1 Introduction

Impairment of the anti‐tumor immune response is a decisive factor allowing for unrestrained cancer growth and progression [1]. It is caused by intercellular interactions in the tumor microenvironment (TME), majorly mediated by signals provided by soluble mediators and microvesicles [1, 2]. These mediators encompass not only cytokines and growth factors, but also bioactive lipids [3, 4], which include cleavage products of phospholipids, such as lysophosphatidic acids, polyunsaturated fatty acids (PUFAs) and PUFA‐derived prostaglandins and other eicosanoid metabolites of arachidonic acid (AA). Except for some prostaglandins, in particular prostaglandin E_2_ [5, 6], the relevance of lipid mediators for immune suppression is poorly understood. This applies especially to nonmetabolized PUFAs, even though high levels have been found in the TME, where they were hypothesized to exert pro‐tumorigenic functions [7]. Indeed, AA levels in malignant ascites have been associated with a short relapse‐free survival (RFS) of ovarian carcinoma (OC) [8], suggesting that this PUFA deserves particular attention in the context of the intercellular communication network of the TME.

While the functions of AA metabolites in the TME have been addressed in a plethora of studies [5], the role of nonmetabolized AA in suppressing anti‐tumor immune surveillance is poorly understood. AA, like other PUFAs, have been reported to interact with different cellular receptors, including the membrane‐bound G‐protein‐coupled free fatty acids receptors (FFAs) [9] and the nuclear receptor PPARβ/δ [7, 10]. It is, however, unlikely that PPARβ/δ mediates the adverse effect of AA on OC RFS, as the potent PPARβ/δ agonist linoleic acid is the dominant PUFA in ascites, but appears not associated with clinical outcome [7].

Other potential targets of nonmetabolized AA include intracellular signal transduction proteins, such as protein kinase C [11, 12, 13, 14, 15], the MAP kinases p38 and JNK [16, 17, 18], and the NADPH oxidase NOX‐2 [19, 20]. We have recently identified a signaling pathway including Ca^2+^ → CAMK2 → ASK1 → p38δ/α → Rho GTPases/HSP27 that is activated by nonmetabolized AA in macrophages, and is linked to impaired actin filament organization, diminished actin‐driven macropinocytosis and enhanced release of exosome‐like vesicles [21], which may partly explain the association of AA with a short RFS of OC.

Arachidonic acid has also been described to exert direct effects by its insertion into cellular membranes, leading to altered mechanical properties affecting the function of membrane channels [20] and transmembrane receptors [22]. PUFAs are also known to be incorporated into lipid rafts, which compartmentalize signal‐transduction‐mediating protein kinases [23, 24], for example members of the of the SRC family [25].

One of the most abundant cell types in the TME, including OC ascites, is the tumor‐associated macrophage (TAM) [26]. TAMs exert a pivotal role in the TME, where they promote tumor progression and immune suppression, and consequently are associated with a poor clinical outcome in different cancer entities [27], including ovarian carcinoma tissue [28] and ascites [29]. TAMs are derived from both resident macrophages and blood monocytes both of which are reeducated by the TME to adopt a spectrum of phenotypes [29, 30, 31, 32]. TAMs from OC ascites, for example, consist of populations with fundamentally different phenotypes and clinical relevance. Thus, CD163^high^ and CD163^high^CD206^high^ TAMs express tumor‐promoting genes and are associated with a short RFS, whereas CD163^low^CD206^low^ TAMs express immune stimulatory genes and are linked to a favorable clinical course [33]. Consistent with these findings, the expression of genes linked to interferon (IFN) signaling in TAMs was associated with prolonged RFS [34]. Furthermore, OC ascites inhibited NFκB activation and induction of the NFκB target gene *IL12B* in macrophages, leading to diminished secretion of T‐cell‐stimulatory IL‐12 [34, 35].

It remains unknown whether the observations summarized above are linked to the potentially detrimental signaling functions of PUFAs in the OC TME. In this study, we have addressed this question in an experimental model of primary monocyte‐derived macrophages (MDMs) exposed to PUFAs found in OC ascites with the aim to investigate the potential role of these lipid mediators in suppressing the immune stimulatory function of macrophages in the TME.

## 2 Materials and methods

### 2.1 Isolation and culture of monocyte‐derived macrophages

Mononuclear cells were isolated by Ficoll density gradient centrifugation from Leukoreduction System (LRS) chambers with leucocytes from healthy adult volunteers kindly provided by the Center for Transfusion Medicine and Hemotherapy at the University Hospital Gießen and Marburg. The collection and analysis of human material were approved by the ethics committee of Philipps University Marburg (reference number 205/10 Amendment 5) in accordance with the standards of the Declaration of Helsinki and with the understanding and written consent of each donor. Monocytes were seeded at approximately 2 × 10^7^ cells per 100 mm dish, 2.5 × 10^6^, 1 × 10^6^ or 0.5 × 10^6^ cells per well in 6‐well, 12‐well or 24‐well, respectively. The adherent cells were washed twice with 10 mL of PBS and differentiated for 6 days in RPMI1640 (Life Technologies, Darmstadt, Germany) supplemented with 5% human AB serum (Sigma‐Aldrich, Taufkirchen, Germany), 1 mm sodium pyruvate (Sigma‐Aldrich, Taufkirchen, Germany). Under these culture conditions, the macrophage‐specific markers CD206 (MRC1) and HLA‐DR were > 95% as determined by flow cytometry. Twenty‐four hours prior to any experiment, the medium was replaced with serum‐free medium for serum starvation.

### 2.2 Treatment of MDMs with cytokines

IFNβ, IFNγ, and IL‐6 were obtained from Biomol (Hamburg, Germany) and used at concentrations of 20 ng·mL^−1^, 40 mg·mL^−1^, and 20 ng·mL^−1^, respectively, in all experiments. Ultrapure lipopolysaccaride (LPS; from *Escherichia coli*) was purchased from InvivoGen (Toulouse, France) and used at 100 ng·mL^−1^. Recombinant human TGFβ1 was purchased from Bio‐Techne (Wiesbaden, Germany) and used at 35 ng·mL^−1^.

### 2.3 Small‐molecule compounds

Polyunsaturated fatty acids, deuterated arachidonic acid (AA‐d8), 5,8,11,14‐eicosatetraynoic acid (ETYA), and Triacsin C were obtained from Cayman Chemicals (Hamburg, Germany), Ruxolitinib from InvivoGen, BIRB796 (Doramapimod) from Biomol, SB203580 from Biozol (Eching, Germany), cholesterol‐methyl‐β‐cyclodextrin from Sigma‐Aldrich.

### 2.4 RT‐qPCR

RNA isolation, cDNA preparation, and qPCR analyses were performed as described [8, 36], using *RPL27* for normalization. Raw data were evaluated by the Cy0 method [37]. Primer sequences are listed in Table S1.

### 2.5 RNA‐sequencing

Total RNA was isolated from MDMs using the NucleoSpin RNA II kit (740955.250; Macherey‐Nagel, Düren, Germany). RNA quality was assessed using the Experion RNA StdSens Analysis Kit (Bio‐Rad, Hercules, CA, USA). RNA‐sequencing (RNA‐Seq) libraries were constructed using the ‘Lexogen Quantseq 3′mRNA‐seq Library Prep Kit FWD for Illumina’ (Lexogen, Vienna, Austria) in combination with the ‘Lexogen UMI Second Strand Synthesis Module for QuantSeq FWD (Illumina, Read 1)’, according to the manufacturer's instructions. Quality of sequencing libraries was controlled on a Bioanalyzer 2100 using the Agilent High Sensitivity DNA Kit (Agilent, Waldbronn, Germany). Pooled sequencing libraries were quantified and sequenced on the Illumina NextSeq550 platform with 75 base single reads.

Data were aligned to the human genome retrieved from Ensembl 96 [38] using star (version STAR_2.6.1d) [39]. Gene read counts were established as read count within merged exons of protein‐coding transcripts (for genes with a protein gene product) or within merged exons of all transcripts (for noncoding genes) and CPM (counts per million). All genomic sequence and gene annotation data were retrieved from Ensembl release 96, genome assembly hg38. RNA‐Seq data were deposited at EBI ArrayExpress (accession numbers E‐MTAB‐10866, E‐MTAB‐10867, E‐MTAB‐10868).

RNA‐Seq data for TAMs have been published in previous studies [8, 33] and were deposited at EBI ArrayExpress (accession numbers E‐MTAB‐4162, E‐MTAB‐5498).

### 2.6 IL‐12 ELISA

Monocyte‐derived macrophages were incubated with 100 ng·mL^−1^ LPS for 24 with or without preincubation with 50 µm AA or ETYA 50 µm for 30 min. IL‐12/p40 concentrations were measured in in cell‐free supernatants from cultured cells using a commercial ELISA kit (430706; Biolegend, San Diego, CA, USA) according to the instructions of the manufacturer.

### 2.7 Immunofluorescence staining of STAT1 and STAT3

Monocyte‐derived macrophages cultured on cover slips were treated with IFNγ or IL6 for 30 min after preincubation with AA 50 µm for 30 min. Cells were fixed with 3.7% paraformaldehyde for 10 min at room temperature and washed three times with PBS. Fixed cells were permeabilized with 0.2% Triton‐X100 for 5 min at room temperature and blocked with bovine serum albumin (BSA) blocking buffer (5% BSA in PBS+ 0.1% Tween 20) for 30 min at room temperature. The cells were stained with anti‐STAT1 or anti‐STAT3 antibody diluted in blocking buffer overnight at 4 °C and secondary antibody diluted in blocking buffer for 1 h at room temperature in the dark. Coverslips were mounted on to glass slides using a drop of mounting medium with 4′,6‐diamidin‐2‐phenylindol (DAPI; VEC‐H‐1200; Vector, Burlingame, CA, USA) and sealed with nail polish. Images were acquired by confocal microscopy (Leica SP8; Leica Microsystems, Wetzlar, Germany).

### 2.8 Immunoblotting and quantification

Immunoblotting was performed according to standard protocols. Shortly, MDMs were washed three times with ice‐cold PBS and lysed in RIPA (10 mm Tris–HCl pH 7.5, 150 mm NaCl, 1% v/v NP40, 1% w/v sodium deoxycholate, 1 mm EDTA) plus protease inhibitor mix (1 : 1000; Sigma), and phosphatase inhibitor mix (50 mm β‐glycerophosphate, 1 mm sodium orthovanadate, 10 mm sodium fluoride and 5 mm sodium pyrophosphate). Proteins were separated by sodium dodecyl sulfate‐polyacrylamide gel electrophoresis (SDS/PAGE) and then transferred to polyvinylidine difluoride membranes (0.45 μm; Carl Roth, Karlsruhe, Germany). Blots were blocked with 3% BSA in PBS with 0.1% Tween 20 for 60 min at room temperature, incubated with primary antibodies at 4 °C overnight, washed three times with PBS with 0.1% Tween 20 and then incubated for 1 h with HRP‐conjugated secondary antibody. After washing, Imaging and quantification were carried out using the ChemiDoc MP system and image lab software version 5 (Bio‐Rad). Phosphoform signals were normalized against the respective protein signals. The following antibodies were used: p‐p38 (T180/Y182; #4511; Cell Signaling, Frankfurt, Germany); p38 (#9228; Cell Signaling), p‐STAT1 (T701; #612132; BD Bioscience, Franklin Lakes, NJ, USA); Stat1 (9172, Cell Signaling); p‐STAT3 (Y705; #9145; Cell Signaling); STAT3 (#9139; Cell Signaling); p‐JAK1 (T1034/1035; #66245; Cell Signaling); JAK1 (50996; Cell Signaling); p‐JAK2 (Y1007/1008; #8082S; Cell Signaling); JAK2 (#3230; Cell Signaling); Flotillin‐1 (#74566; Santa Cruz Technologies, Dallas, TX, USA); CD71 (#65882; Santa Cruz); IκB‐α (#371; Santa Cruz); IκBβ (#8635; Cell Signaling); β‐actin (#A5441; Sigma); Phospho‐SMAD2 (Ser465/467, #3108S; Cell Signaling); SMAD2 (#sc‐393312; Santa Cruz); GAPDH (#G9545; Sigma), α‐rabbit IgG HRP‐linked AB (#27; Cell Signaling) and α‐mouse IgG HRP‐linked AB (#32; Cell Signaling).

### 2.9 Isolation of lipid rafts

Monocyte‐derived macrophages were cultured as described previously. Isolation of lipid rafts was carried out according to a previously described method [40]. Shortly, 8 × 10^7^ cells (four 100 mm diches) were treated with 50 µm AA, ETYA or solvent for 1 h, rinsed three times with ice‐cold PBS and harvested by gentle scraping in 1.4 mL ice‐cold membrane raft isolation buffer (10 mm Tris‐HCl pH 7.4, 150 mm NaCl, 5 mm EDTA, 1 mm Na_3_VO_4_, 1% Triton X‐100 and protease inhibitor). Cells were incubated for 1 h on ice followed by 15 strokes in a Dounce homogenizer. Nuclei and unbroken cells were pelleted by centrifugation at 200 × ***g*** for 8 min and 1 mL of the supernatant was mixed with 1 mL of 85% sucrose (w/v), transferred to Ultra‐Clear centrifuge tubes (#344059; Beckmann Coulter, Krefeld, Germany), sequentially overlayed with 5 mL of 35% sucrose (w/v) and 3.5 mL of 5% sucrose (w/v). and centrifuged at 248 000 × ***g*** (SW41 Ti; Beckman Coulter) for 18 h at 4 °C. Eleven 1‐mL fractions from the top were collected from each gradient. Thirty microliters of each fraction were analyzed by immunoblotting. Fraction #4 was used for proteomic analysis.

### 2.10 Proteomic analysis of lipid rafts

Proteomic analysis of lipid raft samples in biological pentuplicate was performed by GeLC/MS2 (in gel digest/liquid chromatography/tandem mass spectrometry) as described [33]. Peptide/spectrum matching and label‐free quantification was performed using the maxquant suite of algorithms (v. 1,6,17,0) [41, 42, 43] against the human uniprot database [44] (canonical and isoforms; 194 237 entries; downloaded 2021/02/08). Instrument parameters were extracted and summarized using marmoset [45] and along with the relevant maxquant configuration are included in Supplemental Methods. The data have been deposited with the ProteomeXchange Consortium via the PRIDE partner repository [46] with the dataset identifier PXD028434. Downstream data processing was performed using the r (http://www.r‐project.org/index.html) and limma [47] based package autonomics (https://bioconductor.org/packages/autonomics). Data were filtered for completeness, logarithmized, quantile normalized and consistently missing nondetects imputed. Limma‐based linear modeling for detection of differentially detected protein features used replicates as an additional covariate.

### 2.11 Lipid analysis of lipid rafts by LC‐MS

Quantification of arachidonic acid was performed as described previously [7] with slight modifications. Membrane samples were spiked with 10 µL AA‐d8 (10 ng·mL^−1^), acidified with 10 µL acidic acid (10%) and extracted with diisopropylether. The upper phase was evaporated and the sample resuspended in 100 µL solvent A [water/acetonitrile (70 : 30) with 0.02% formic acid]. Analysis was done by LC‐MS/MS on an Agilent 1290 HPLC coupled to a QTrap 5500 mass spectrometer (AB Sciex, Framingham, MA, USA). Samples were separated on a Synergi reverse‐phase C18 column (2.1 × 100 mm; Phenomenex, Aschaffenburg, Germany) using a gradient of 60–100% solvent B (acetonitrile/isopropyl alcohol, 50 : 50) over 6 min. The column was re‐equilibrated at 60% solvent B for 3 min. The flow rate was 0.3 mL·min^−1^. Compounds were detected in multiple reaction monitoring mode (transitions: AA 303‐>259, AA‐d8 311‐>267). For quantification, a 9‐point calibration curve was used. Data analysis was performed using analyst 1.7.2 and multiquant 2.1.1 (AB Sciex).

For lipidomic analysis of PUFAs, a MSMSALL workflow was applied as described elsewhere [48]. Two hundred microlitre of membrane sample was mixed with 1.2 mL methanol, 1 mL water, 10 µL SPLASH^®^ LIPIDOMIX^®^ Mass Spec Standard (Avanti Lipids, Alabaster, AL, USA) and extracted with 4 mL diisopropylether. The upper phase was evaporated and the sample resuspended in 200 µL HPLC solvent [methanol/dichlormethane (50 : 50) with 5 mm ammonium acetate]. One hundred microlitre of the sample was automatically infused into the ESI source, equipped with a 65 µm electrode using an Agilent 1290 HPLC, provided with NanoViper tubings (ID 50 µm; Thermo Scientific, Waltham, MA, USA) with a flow rate of 7 µL·min^−1^. Negative ion scans were performed using a TripleTOF™ 5600+ (AB Sciex) controlled by Analyst^®^ TF 1.7.1 software with activated MS/MSALL mode. The MS/MSALL workflow consisted of a TOF MS scan from *m/z* 400–1000 followed by sequential acquisition of 600 MSMS spectra with a step size of 1.001 Da, measuring across *m/z* 100–1000. The total time for one MS/MSALL acquisition was around 8 min. The acquired data were processed with lipidview™ 1.3 software (AB SCIEX. Foster City, CA, USA). Mass tolerance was set to 0.05 and minimum S/N to 5. Analyzed lipid species were as follows: phosphatidic acid (PS), phosphatidylcholine (PC), phosphatidyletanolamine (PE), phosphatidylglycerol (PG), phosphatidylinositol (PI), and phosphatidylserine (PS).

### 2.12 Pathway analysis

Reactome pathway analysis [49] was performed using the online tool of the Gene Ontology Resource website at http://geneontology.org.

### 2.13 Statistical analysis

Comparative data were statistically analyzed by paired Student's *t* test (two‐sided, equal variance). Significance levels are indicated as ****, ***, ** and * for *P* < 0.0001, *P* < 0.001, *P* < 0.01 and *P* < 0.05, respectively.

## 3 Results

### 3.1 Suppression of cytokine‐induced genes in CD163^low^CD206^low^ TAMs and AA‐treated MDMs

Analysis of an RNA‐Seq dataset of 29 TAMs samples from OC ascites identified *n* = 1160 protein‐coding genes whose expression was inversely correlated with the mRNA levels of *CD163* and *CD206/MRC1* (Spearman < −0.5; Table S2) and hence associated with a poor clinical outcome [33]. Reactome pathways enrichment analysis [49] of these genes (Table 1) yielded ‘Cytokine signaling in immune system’ at the most significant term (*n* = 101 gene; FDR = 4 × 10^−8^), followed by specific signal transduction pathways (IFN, TNF, TLR4; *n* = 16–33 genes; FDR < 0.03), which are also included in the former term.

**Table 1:** Reactome pathways enrichment analysis of genes inversely correlated with *CD163/CD206* expression in TAMs. Analysis of RNA‐Seq data for TAMs from 29 OC patients yielded *n* = 1193 genes for Spearman rho < −0.5 and nominal *P* < 0.05. The table shows the top 10 hits (query genes in pathway > 15; fold enrichment > 2; FDR < 0.05).

Reactome pathway	Query genes in pathway (*n*)	Fold enrichment	FDR
Cytokine signaling in immune system	101	2.14	4 × 10^−8^
Interferon signaling	33	2.93	0.0001
Interferon gamma signaling	21	4.02	0.0002
HSP90 cycle for steroid receptors	16	5.06	0.0003
TNFR2 noncanonical NFκB pathway	18	3.17	0.0097
Ub‐specific processing proteases	27	2.30	0.0210
Death receptor signaling	21	2.59	0.0290
MyD88‐independent TLR4 cascade	16	2.90	0.0293
TRIF‐mediated TLR4 signaling	16	2.90	0.0304
Deubiquitination	33	2.04	0.0322

**Fig. 1:**
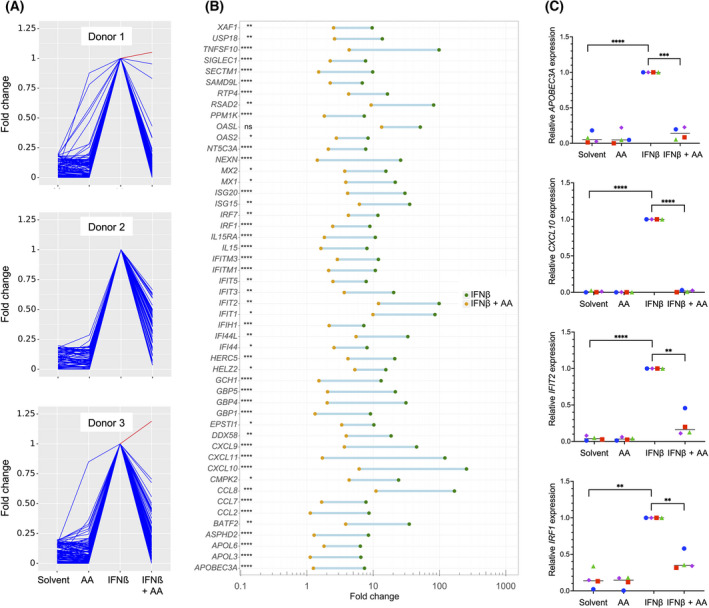
Impact of arachidonic acid (AA) on the transcriptome of IFNβ‐stimulated monocyte‐derived macrophages (MDMs). MDMs were pretreated with 50 µm AA or solvent for 30 min prior to stimulation with IFNβ for 3 h followed by RNA‐Seq analysis. (A) RNA‐Seq results for the top genes induced by IFNβ (fold cahnge ≥ 5 for IFNβ versus solvent; counts per million ≥ 5 for IFNβ‐stimulated cells; *n* = 3 donors). Data were normalized for INFβ‐stimulated cells, and data points were connected by lines for improved visualization. Blue: IFNβ‐induced genes repressed by AA; red: IFNβ‐induced genes upregulated by AA. (B) IFNβ‐induced genes showing the strongest repression by AA (top 50 IFNβ induced genes; FDR < 0.05 for IFNβ versus IFNβ plus AA). The green and orange data points show the mean (*n* = 3) induction values for IFNβ and IFNβ plus AA, respectively. (C) Validation of RNA‐Seq results by RT‐qPCR for *APOBEC3A, CXCL10, IFIT2* and *IRF1* using *RPL27* as the normalizer. Cy0 values are expressed relative to IFNβ‐stimulated cells for *n* = 4 donors (represented by different symbols). Statistical significance was analyzed by paired *t* test (*P < 0.05; **P < 0.01; ***P < 0.001; ****P < 0.0001). Horizontal lines indicate the median.

**Fig. 2:**
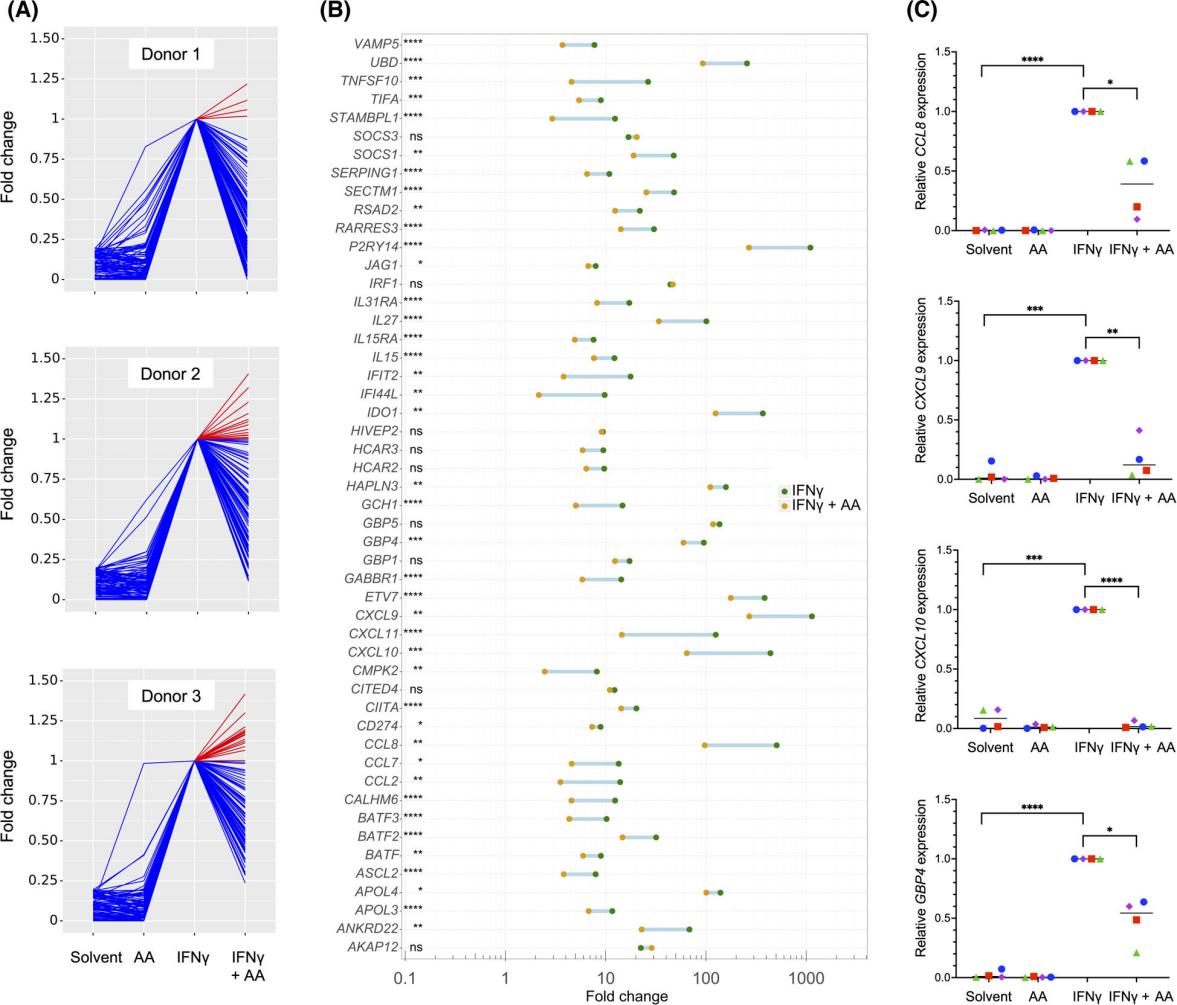
Impact of arachidonic acid (AA) on the transcriptome of IFNγ‐stimulated monocyte‐derived macrophages (MDMs). MDMs were treated and analyzed as in Fig. 1 except that IFNγ was used instead of INFβ. (A) RNA‐Seq results for the top IFNγ‐induced genes (fold change ≥ 5 for IFNγ versus solvent; counts per million ≥ 5 for IFNγ‐stimulated cells; *n* = 3 donors). Data were normalized for INFγ‐stimulated cells, and data points were connected by lines for improved visualization. Blue: IFNγ‐induced genes repressed by AA; red: IFNγ‐induced genes upregulated by AA. (B) IFNγ‐induced genes showing the strongest repression by AA (C) Validation of RNA‐Seq results by RT‐qPCR for *CCL8, CXCL9, CXCL10* and *GBP4*. Cy0 values are expressed relative to IFNγ‐stimulated cells for *n* = 4 donors (represented by different symbols). Statistical significance was analyzed by paired *t* test (*P < 0.05; **P < 0.01; ***P < 0.001; ****P < 0.0001). Horizontal lines indicate the median.

**Fig. 3:**
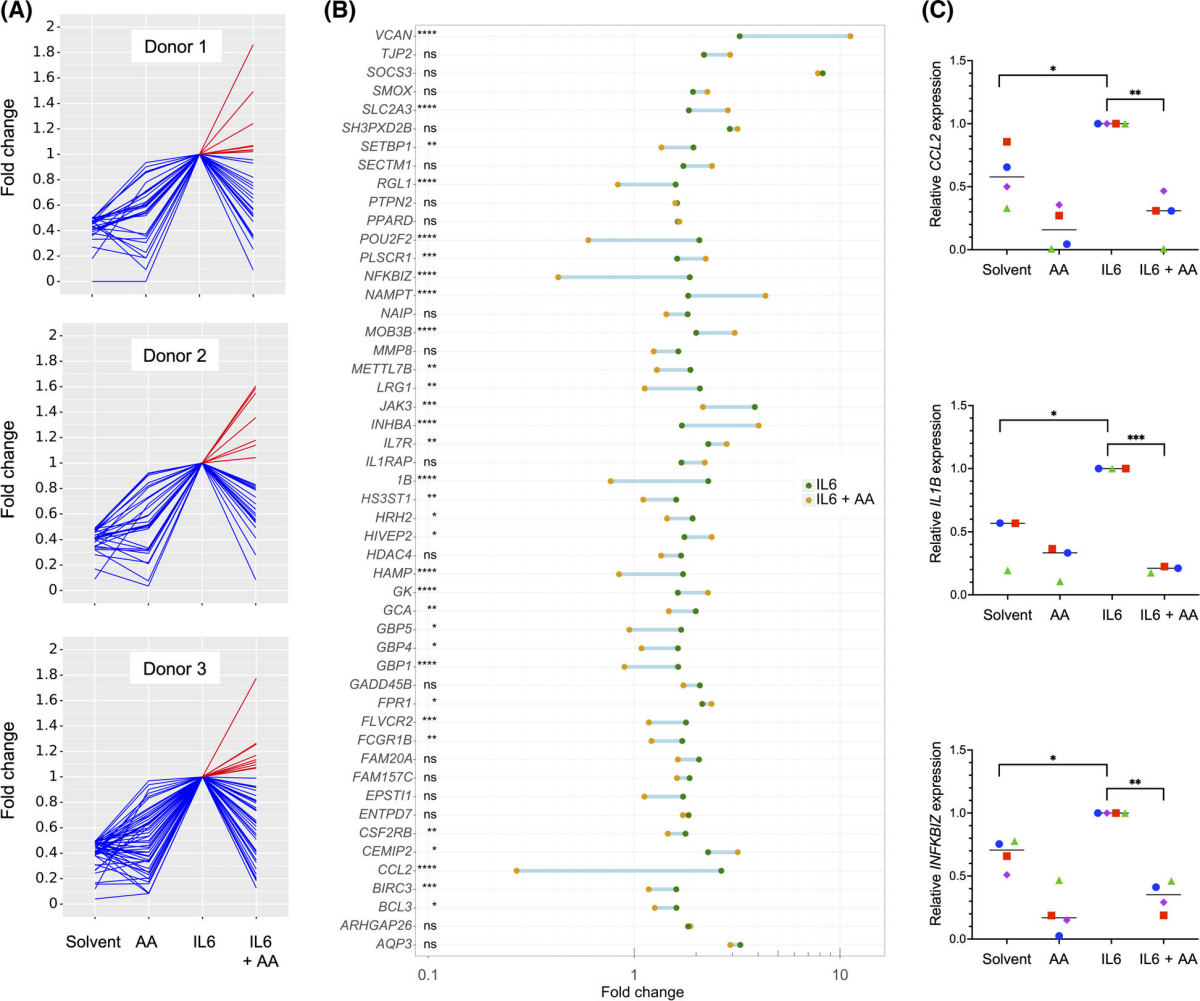
Impact of arachidonic acid (AA) on the transcriptome of IL6‐stimulated monocyte‐derived macrophages (MDMs). MDMs were treated and analyzed as in Fig. 1 except that IL‐6 was used instead of IFNβ. (A) RNA‐Seq results for the top IL‐6‐induced genes (fold change ≥ 2 for IL‐6 versus solvent; counts per million ≥ 5 for IL‐6 stimulated cells; *n* = 3 donors). Data were normalized for IL‐6‐stimulated cells, and data points were connected by lines for improved visualization. Blue: IL‐6‐induced genes repressed by AA; red: IL‐6‐induced genes upregulated by AA. (B) IL‐6‐induced genes showing the strongest repression by AA (C) Validation of RNA‐Seq results by RT‐qPCR for *CCL2, IL1B,* and *IFNKBIZ*. Cy0 values are expressed relative to IL‐6‐stimulated cells for *n* = 4 (*CCL2, IFNKBIZ*) or *n* = 3 (*IL1B*) donors (represented by different symbols). Statistical significance was analyzed by paired *t* test (*P < 0.05; **P < 0.01; ***P < 0.001; ****P < 0.0001). Horizontal lines indicate the median.

The data in Table 1 also suggest a major impact of the OC TME on cytokine‐triggered signal transduction in macrophages. To address the question whether PUFAs in the TME may play a role in this context, we investigated the impact of AA on the transcriptional responses to IFNβ, IFNγ and IL‐6 in primary MDMs. As illustrated by Figs 1A, 2A, and 3A, AA produced a strong inhibitory effect on the cytokine responses (blue lines) with minor donor‐dependent differences (RNA‐Seq data in Tables S3–S5). The top 50 cytokine‐induced genes (strongest repression by AA) are depicted for IFNβ, IFNγ, and IL‐6 in Figs 1B, 2B, and 3B, respectively. RNA‐Seq results were verified by RT‐qPCR, as shown in Fig. 1C for the IFNβ target genes *APOBEC3A, CXCL10, IFIT2,* and *IRF1*, in Fig. 2C for the IFNγ target genes *CCL8, CXCL9, CXCL10,* and *GBP4* and in Fig. 3C for the IL‐6 target genes *CCL2, IL1B,* and *INFKBIZ*. These results clearly indicate that AA suppresses the target genes of pro‐inflammatory cytokines that are known to activate different intracellular signal transduction pathways.

### 3.2 Suppression of JAK‐STAT signaling by AA and other PUFAs

**Fig. 4:**
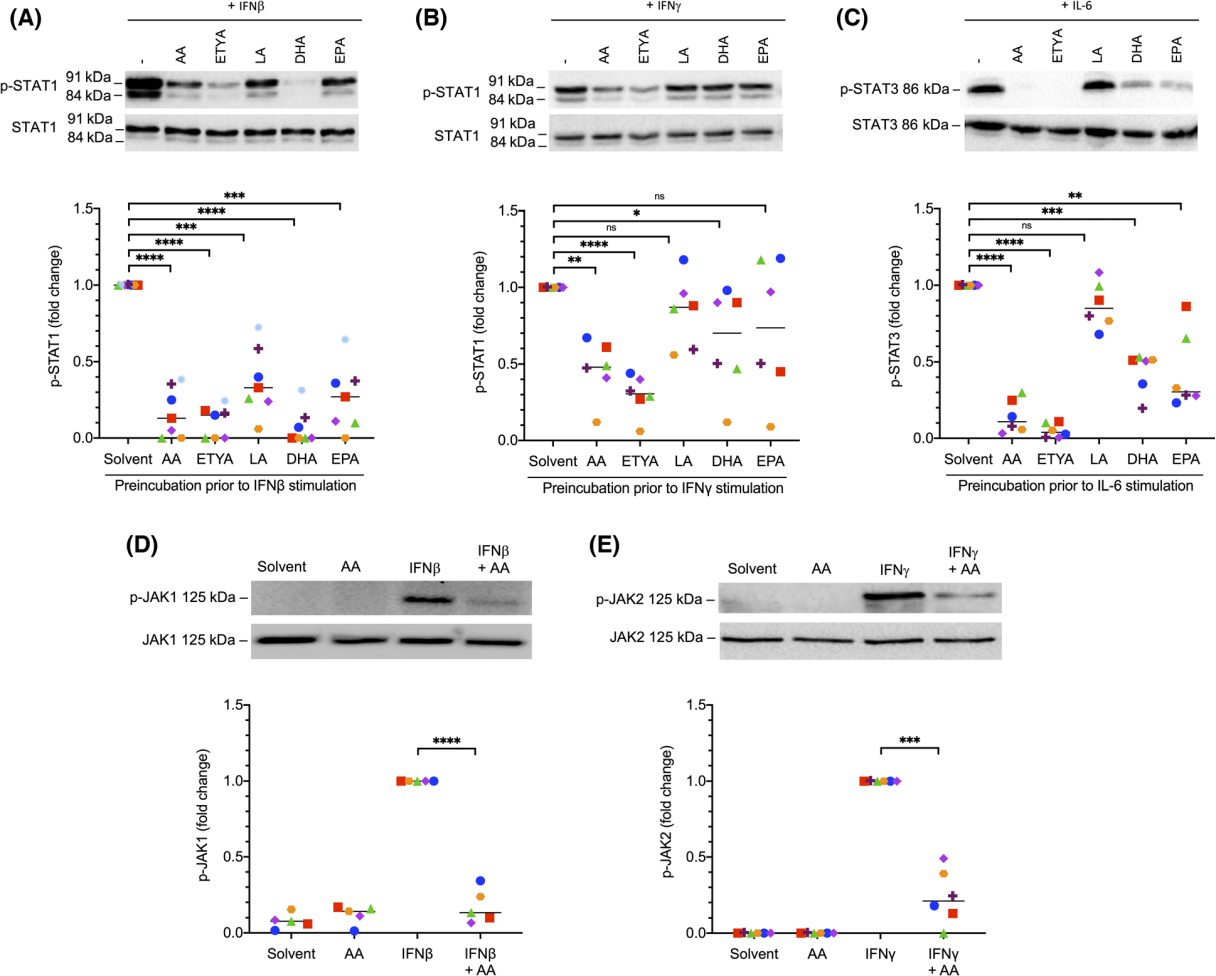
Inhibition of cytokine‐induced STAT and JAK signaling in monocyte‐derived macrophages (MDMs) by polyunsaturated fatty acids (PUFAs). (A) Inhibition of IFNβ‐induced phosphorylation of STAT1 (Y701) by different PUFAs. The p‐STAT1 antibody recognizes both the STAT1α and β isoforms. (B) Inhibition of IFNγ‐induced phosphorylation of STAT1 by different PUFAs. (C) Inhibition of IL‐6 induced phosphorylation of STAT3 (Y705) by different PUFAs. AA, arachidonic acid; LA, linoleic acid; EPA, eicosapentaenoic acid; DHA, docosahexaenoic acid; ETYA, 5,8,11,14‐eicosatetraynoic acid. (D) Inhibition of IFNβ‐induced phosphorylation of JAK1 (Y1034/1035) by AA. (E) Inhibition of IFNγ‐induced phosphorylation of JAK2 (Y1007/Y1008) by AA. In each case, MDMs were pretreated with 50 µm of the indicated PUFA for 30 min prior to stimulation with the IFNβ, IFNγ or IL‐6 for 30 min. A representative immunoblot and the quantification of *n* = 7 (A–C) or *n* = 5 (D–E) independent experiments (different donors; indicated by different symbols) are shown in each panel. Statistical significance was analyzed by paired *t* test (*P < 0.05; **P < 0.01; ***P < 0.001; ****P < 0.0001; ns, not significant). Horizontal lines indicate the median.

**Fig. 5:**
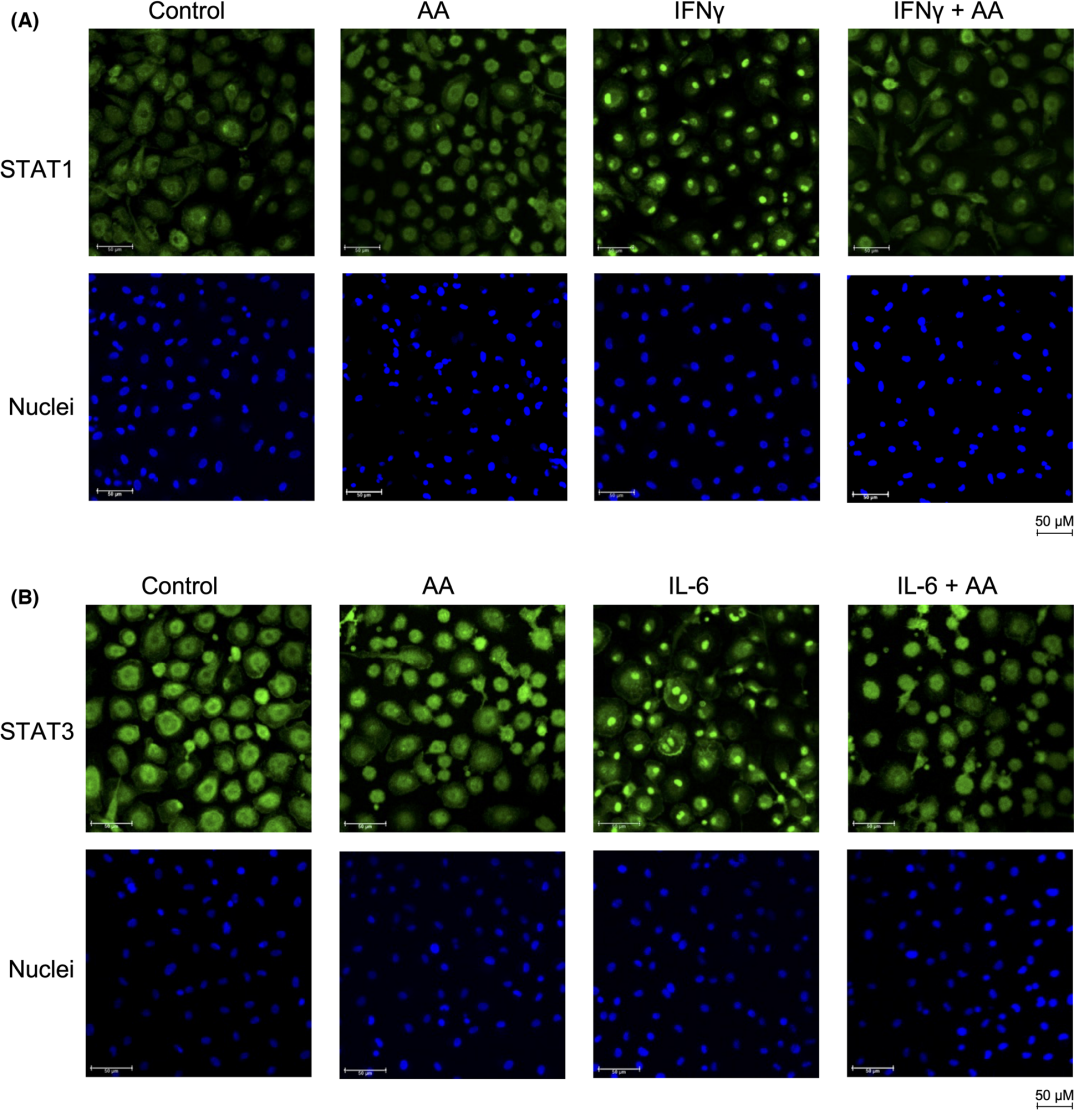
Inhibition of the cytokine‐induced nuclear translocation of STAT1 and STAT3 by arachidonic acid (AA). (A) Monocyte‐derived macrophages (MDMs) were pretreated with 50 µm AA or solvent for 30 min prior to stimulation with IFNγ for 30 min as in Fig. 4 and the subcellular localization of STAT1 was analyzed by immunofluorescence (green). Nuclei were visualized by staining with 4′,6‐diamidin‐2‐phenylindol (DAPI). (B) Stimulation of MDMs with IL6 and staining of STAT3 as in panel A. The figure shows representative images. The experiments were performed with three different donors, which all showed a > 90% inhibition of the nuclear translocation of STAT1 and STAT3, respectively. Scale bars indicate 50 µm.

To understand the regulation of cytokine signaling by AA in more detail, we analyzed the activation of proteins downstream of the cytokine‐bound receptors, that is, JAK1 and STAT1 for IFNβ, JAK2, and STAT1 for IFNγ, and STAT3 for IL‐6. All tested PUFAs inhibited phosphorylation of STAT1 on Tyr‐701 triggered by IFNβ (Fig. 4A and Fig. S1) or IFNγ (Fig. 4B) and phosphorylation of STAT3 on Tyr‐705 triggered by IL‐6 (Fig. 4C), albeit with differences in the extent, significance and target selectivity of the effects observed for different PUFAs. Consistent with this observation, AA inhibited the cytokine‐induced nuclear translocation of STAT1 and STAT3 mediated by IFNγ (Fig. 5A) and IL‐6 (Fig. 5B), respectively. Overall, the inhibitory effect appeared strongest for AA compared with linoleic acid (LA), eicosapentaenoic acid (EPA), and docosahexaenoic acid (DHA) (Fig. 4A–C), which may be relevant, as only AA is significantly associated with a short RFS of OC [7]. Importantly, inhibition of STAT phosphorylation was similar for both AA and the nonmetabolizable AA analog and dual COX/LOX inhibitor ETYA [50], indicating that the observed effects are not dependent on the conversion of AA to other eicosanoids. AA also inhibited phosphorylation of the protein kinases linking IFN receptors to STAT proteins, that is, JAK1 (Fig. 4D) and JAK2 (Fig. 4E), pointing to an inhibitory effect of PUFAs at the initial stages of receptor‐triggered signal transduction.

### 3.3 AA‐mediated inhibition of STAT1 does not involve p38

**Fig. 6:**
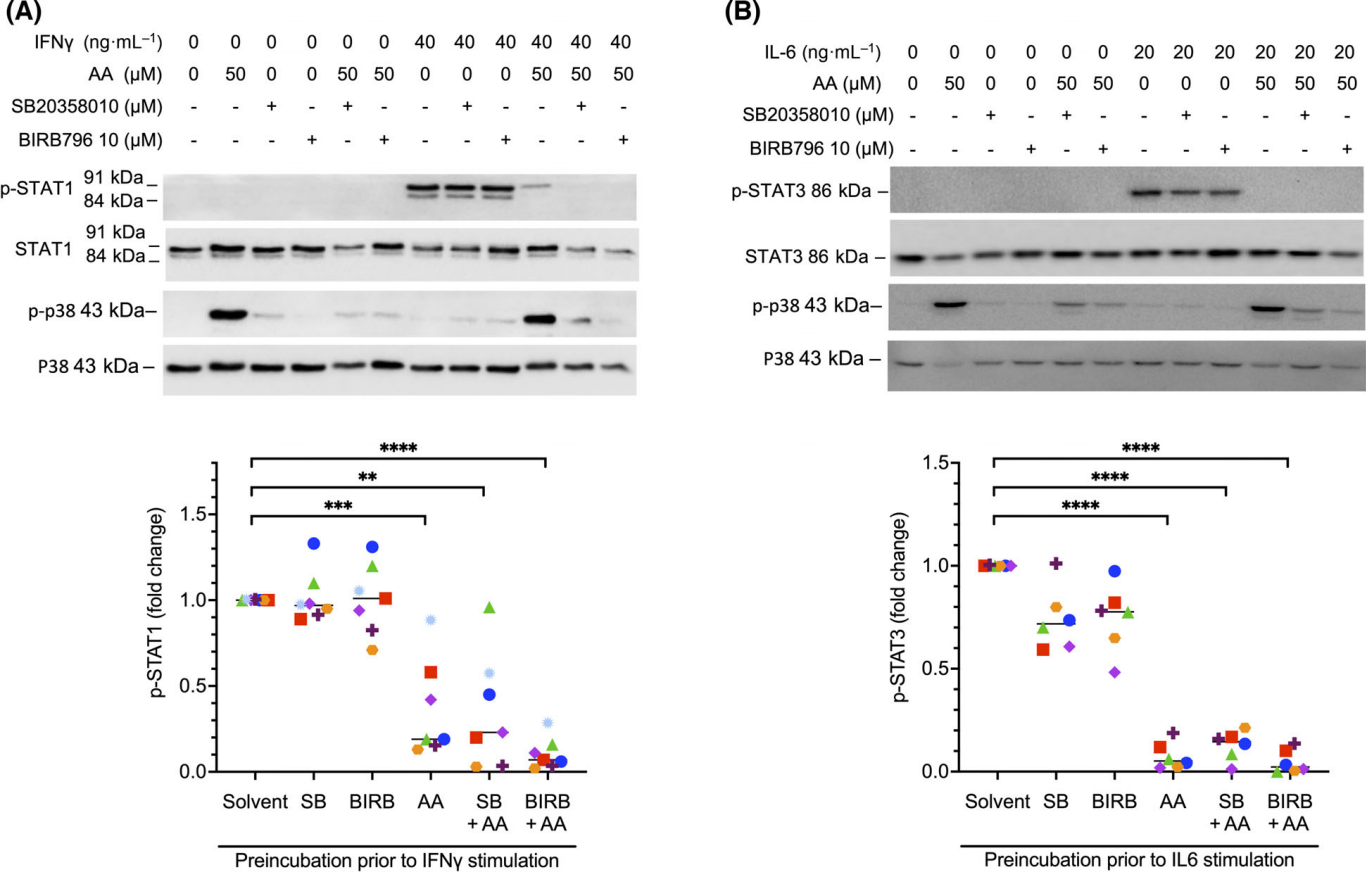
Inhibition of STAT phosphorylation by arachidonic acid (AA) is independent of p38 MAPK. (A) Monocyte‐derived macrophages (MDMs) were stimulated with IFNγ after preincubation with solvent, AA, the p38 inhibitors SB203580 or BIRB796, or combinations of these (details as in Fig. 4). Cell extracts were analyzed for changes in STAT1 (Y701) and p38 (T180/Y182) phosphorylation. The panel shows a representative immunoblot and a quantification for *n* = 7 different donors (represented by different symbols). (B) MDMs were stimulated with IL‐6 after preincubation as in panel A (*n* = 6). Cell extracts were analyzed for changes in STAT3 (Y705) and p38 (T180/Y182) phosphorylation. Statistical significance was analyzed by paired *t* test (**P < 0.01; ***P < 0.001; ****P < 0.0001). Horizontal lines indicate the median.

We have recently reported that AA induces signaling pathway dependent on MAPK13/14 (p38) [21]. We therefore asked whether p38 may be involved in the inhibition of STAT signaling observed in this study. Two lines of evidence strongly argue against such a link. First, maximal effects of AA on p38 phosphorylation were observed at concentrations around 12.5 µm [21], whereas inhibition of STAT1 and STAT3 reaches its maximum at ~ 50 µm (Fig. S2). Second, the p38 inhibitors SB203580 and BIRB796 had no detectable effect on the AA‐mediated inhibition of STAT1 phosphorylation in response to IFNγ or STAT3 phosphorylation triggered by IL‐6 (Fig. 6). Based on these results, we conclude that activation of p38 and inhibition of STAT signaling by AA are unrelated events.

### 3.4 Inhibition of LPS‐induced STAT1 signaling in MDMs by AA

**Fig. 7:**
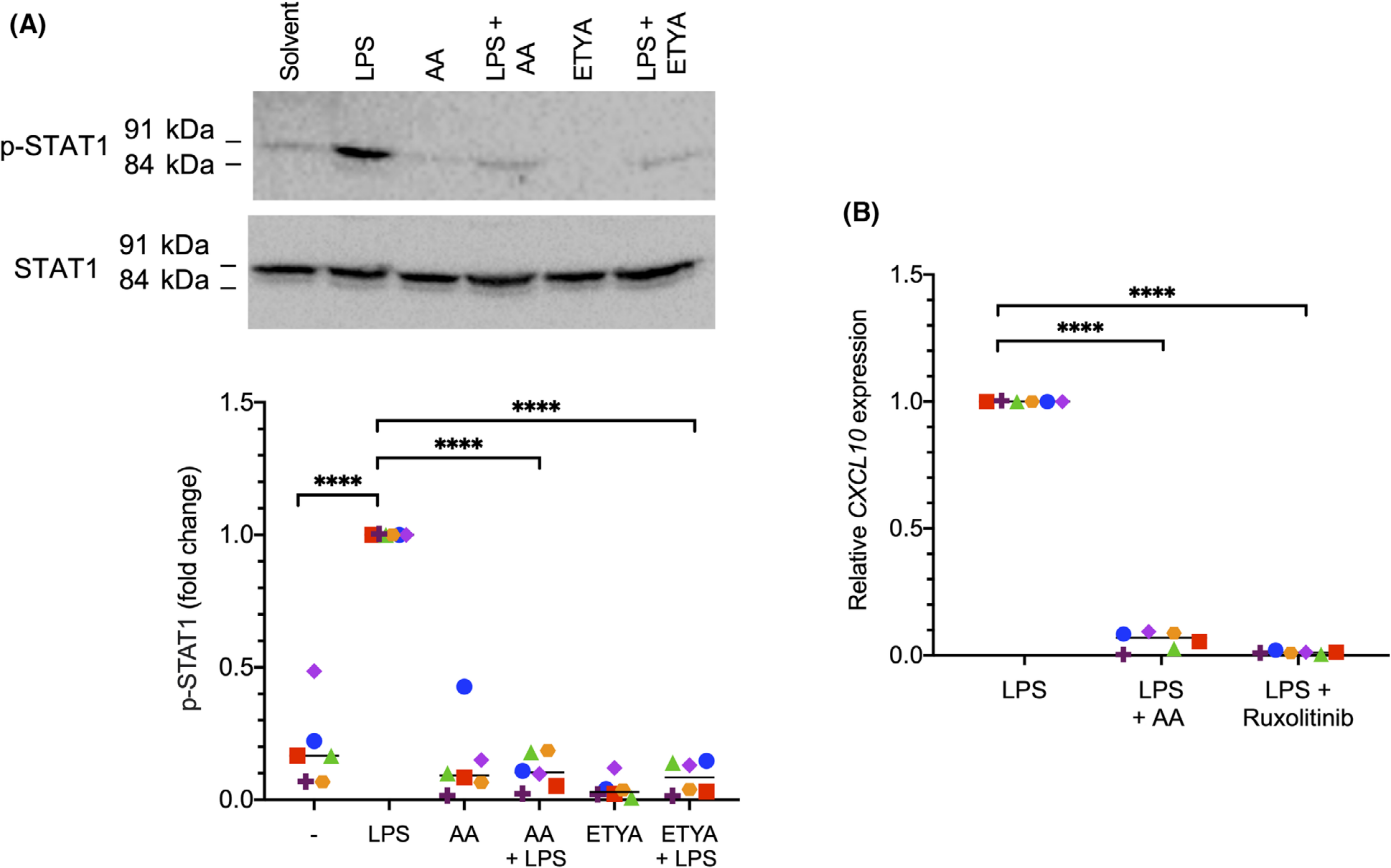
Inhibition of lipopolysaccharide (LPS)‐induced STAT1 signaling in monocyte‐derived macrophages (MDMs) by arachidonic acid (AA). (A) Inhibition of LPS‐induced phosphorylation of STAT1 (Y701) by AA or 5,8,11,14‐eicosatetraynoic acid (ETYA). MDMs were pretreated with 50 µm of AA or ETYA for 30 min prior to stimulation with 100 ng·mL^−1^ LPS for 60 min. A representative immunoblot and quantification of six replicates are displayed. (B) RT‐qPCR analysis showing inhibition of *CXCL10* by AA and verification of *CXCL10* as a STAT1 target gene (*n* = 6 donor; represented by different symbols). MDMs were pretreated with 50 µm AA or the 0.5 µm of the STAT1 inhibitor Ruxolitinib for 30 min prior to stimulation with 100 ng·mL^−1^ LPS for 3 h. Statistical significance was analyzed by paired *t* test (****P < 0.0001). Horizontal lines indicate the median.

It has been described that LPS, among other pathways, also triggers tyrosine phosphorylation of JAK and STAT proteins [51, 52]. We were therefore interested to investigate whether AA is able to interfere with the phosphorylation of STAT1 in this setting. This was indeed the case as documented by the blockade of the LPS‐triggered phosphorylation of STAT1 at Y701 by both AA and ETYA (Fig. 7A). An LPS target gene mainly induced via JAK‐STAT signaling is *CXCL10*, as shown by the complete block of its LPS‐mediated induction by the selective JAK1/JAK2 inhibitor Ruxolitinib [53] in Fig. 7B. AA had a similarly strong inhibitory effect as Ruxolitinib (Fig. 7B), indicating the functional significance of STAT1 in the context of the AA‐mediated repression of LPS‐triggered signaling.

### 3.5 AA‐mediated alterations of lipid rafts as a cause of inhibited JAK‐STAT signaling

**Fig. 8:**
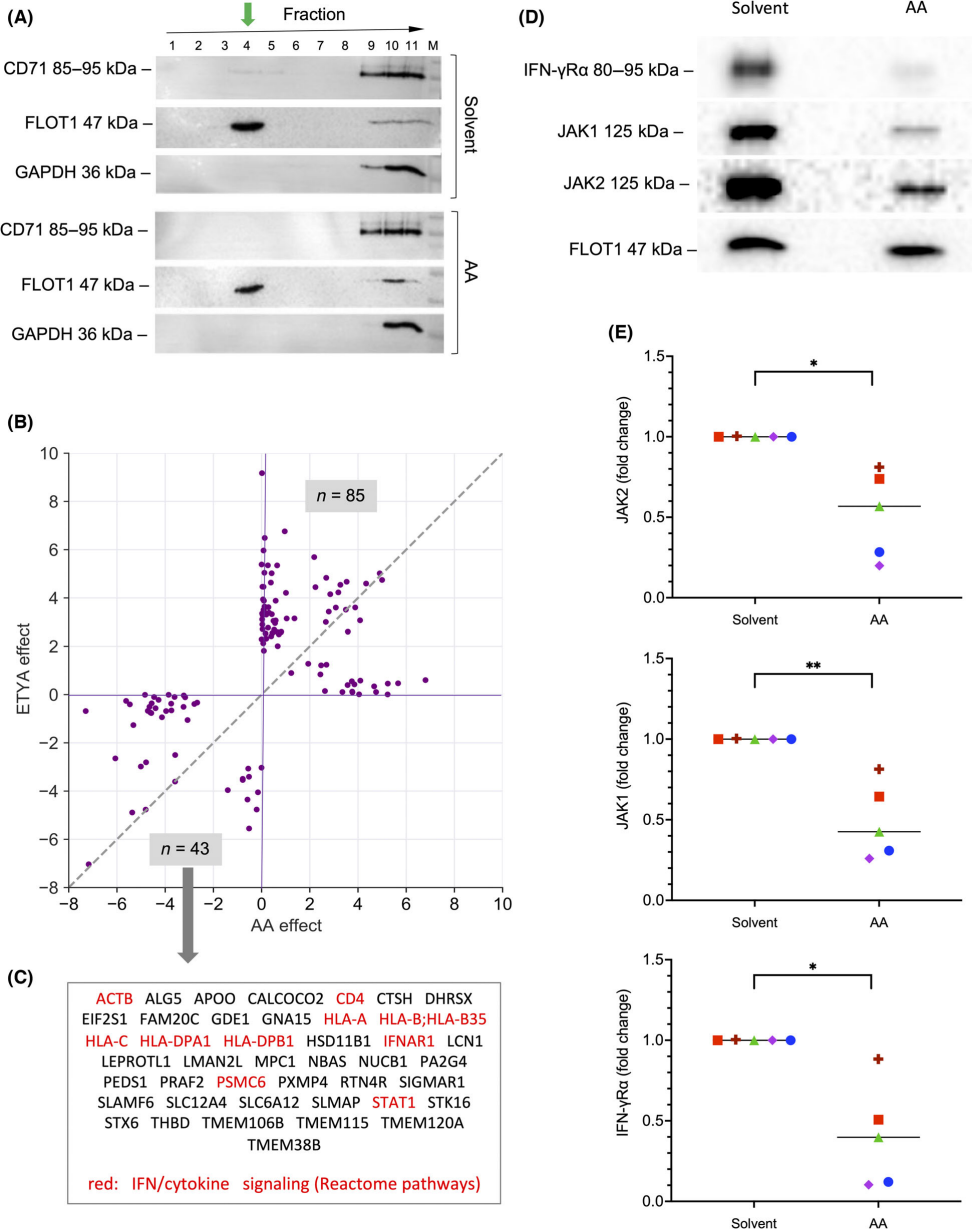
Impact of arachidonic acid (AA) on the composition of lipid rafts in monocyte‐derived macrophages (MDMs). (A) Immunoblot analysis of membrane protein fractions obtained from MDMs after treatment with solvent or 50 µm AA. Membrane components were separated by ultracentrifugation (see Materials and methods for details) and analyzed using antibodies for CD71 (transferrin receptor) as a marker for proteins not enriched in lipid rafts, FLOT1 (flotillin1) as a marker for lipid rafts and GAPDH as a cytosolic marker. The green arrow shows enrichment of FLOT1 in fraction 4, which was used for further analyses. (B) Effects of AA and 5,8,11,14‐eicosatetraynoic acid (ETYA) on the presence of proteins in lipid rafts identified by MS‐based proteomic analysis of fraction 4 proteins. The plot shows all proteins with a |log_2_| difference > 2 (median of *n* = 5 samples) in samples treated with AA or ETYA. Preprocessed data and results of the differential analysis are found in Table S6. (C) Proteins missing in lipid rafts isolated from cells treated with AA or ETYA (bottom left quadrant in panel A). Proteins associated with ‘IFN signaling’ and ‘cytokine signaling’ by Reactome pathway analysis (Table S7) are highlighted in red. (D) Verification of the AA‐triggered displacement of the IFN‐signaling‐associated proteins IFN‐γRα, JAK1 and JAK2 from lipid rafts by immunoblotting. (E) Quantification of *n* = 5 independent experiments as in panel D (five different donors; indicated by different symbols). Statistical significance was analyzed by paired *t* test (*P < 0.05; **P < 0.01). Horizontal lines indicate the median.

As PUFAs can displace proteins from lipid rafts and thereby modulate their signaling function [23, 24], we sought to investigate whether the observed interference by AA with cytokine signaling in MDMs may involve the lipid‐raft localization of receptors and/or receptor‐associated proteins. To address this question, we isolated lipid‐raft‐enriched fractions from MDMs after treatment with 50 µm AA or solvent by sucrose‐gradient ultracentrifugation. Using antibodies for FLOT1 (flotillin1) as a marker for lipid rafts, CD71 (transferrin receptor) as a marker for nonraft plasma membrane proteins and GAPDH as a cytosolic marker we were able to identify highly enriched lipid‐raft‐containing fractions in extracts from both AA‐ and solvent‐treated cells suitable for proteomic analysis (fraction 4 in Fig. 8A). MS‐based proteomic analysis of fraction‐4 proteins from *n* = 5 different MDM samples (Fig. 8B,C; Table S6) identified *n* = 43 proteins that were significantly (FDR < 0.05) decreased (log_2_ difference > 2) in AA and/or ETYA‐treated cells (Fig. 8B), while *n* = 65 proteins were increased. Reactome pathway analysis [49] of the 43 proteins decreased in lipid rafts identified ‘interferon signaling’ as the most significant hit besides ‘cytokine signaling in immune system’ and other related terms (Table S7). Among these proteins are IFNAR1 (the receptor for type I IFNs including IFNβ) and STAT1 (Fig. 8C). In contrast, no significant enrichment was observed with the group of 65 proteins increased in lipid rafts. The AA‐triggered displacement of the IFN‐signaling‐associated proteins from lipid rafts was verified for IFN‐γRα, JAK1 and JAK2 by immunoblotting with *n* = 5 different MDM samples (Fig. 8D,E).

**Fig. 9:**
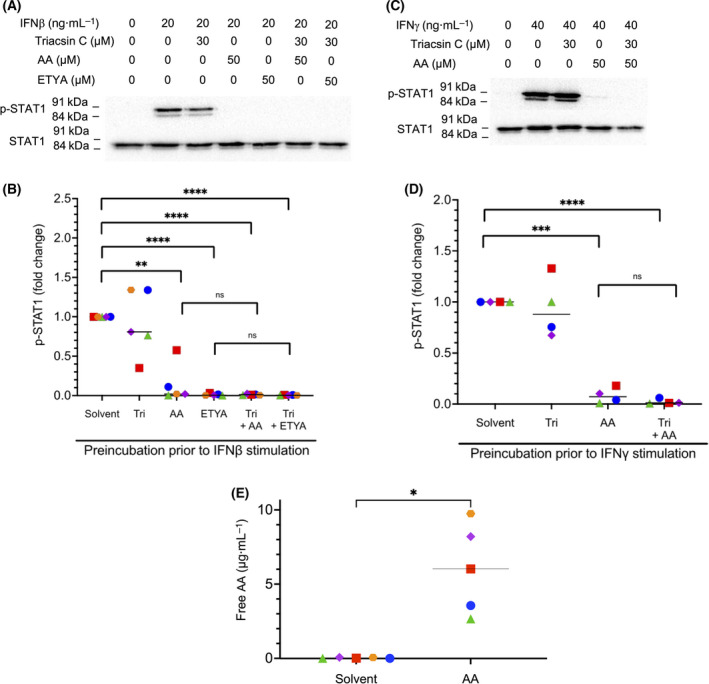
Impact of arachidonic acid (AA) incorporated into phospholipids versus free AA on lipid rafts. (A–D) Analysis of the effect of Triacsin C, an inhibitor of long fatty acyl CoA synthetase, on the AA‐mediated inhibition of STAT1 phosphorylation induced by INFβ (panel A, B) or IFNγ (panel C, D) in monocyte‐derived macrophages (MDMs). Experimental details were as in Fig. 4. Quantifications are shown for of *n* = 5 independent experiments (five different donors; represented by different symbols) in panel B and *n* = 4 donors in panel D. (E) Mass‐spectrometry‐based analysis of concentrations of free AA in *n* = 5 independent preparations of lipid rafts from MDMs treated with solvent or 50 µm AA for 1 h. Statistical significance was analyzed by paired *t* test (*P < 0.05; **P < 0.01; ***P < 0.001; ****P < 0.0001; ns, not significant). Horizontal lines indicate the median.

Next, we asked whether AA incorporated into phospholipids is responsible for the observed inhibition of cytokine signaling. We addressed this question by analyzing the effect of Triacsin C, an inhibitor of long fatty acyl CoA synthetase. As depicted in Fig. 9A–D, Triacsin C did not counteract the AA‐mediated inhibition of STAT1 phosphorylation induced by INFβ (Fig. 9A,B) or IFNγ (Fig. 9C,D) to any detectable extent in *n* = 5 biological replicates, even though lipidomic analyses showed a significant increase in AA‐containing phospholipids in lipid rafts after 50 µm AA treatment for 1 h (Fig. S3). However, MS‐based analysis of five independent lipid raft samples also revealed a dramatic increase in free AA following AA exposure compared to solvent‐treated cells (Fig. 9E). Taken together, these observations suggest that free AA rather that phospholipid‐bound AA is a crucial determinant of its inhibitory effect on IFN signaling, which is consistent with previously reported findings [23, 24].

**Fig. 10:**
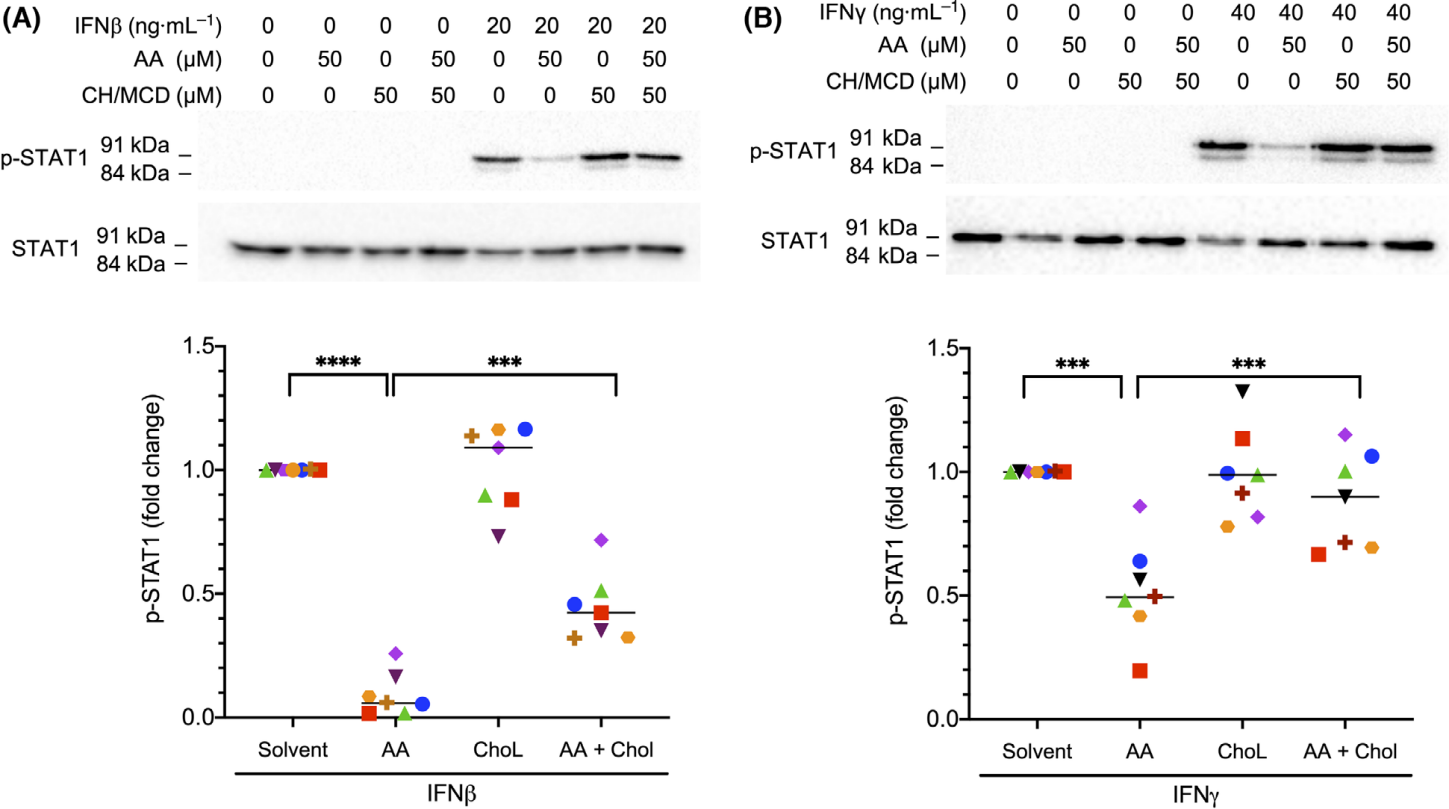
Abrogation of the inhibitory effect of arachidonic acid (AA) on STAT1 phosphorylation by water‐soluble cholesterol/methyl‐β‐cyclodextrin (Ch/MCD). Monocyte‐derived macrophages (MDMs) were pretreated with 50 µm AA for 30 min and 50 µm Ch/MCD prior to stimulation with IFNβ (A) or IFNγ (B) for 30 min. Representative immunoblots and quantification for MDMs from *n* = 7 different donors (represented by different symbols) are shown. Statistical significance was analyzed by paired *t* test (***P < 0.001; ****P < 0.0001). Horizontal lines indicate the median.

Importantly, the inhibitory effect of AA on IFNβ‐ or IFNγ‐triggered STAT1 phosphorylation was largely abrogated by water‐soluble cholesterol (complex of cholesterol with methyl‐β‐cyclodextrin; Fig. 10). Cholesterol/methyl‐β‐cyclodextrin (Chol/MCD) prevents the displacement of cholesterol from lipid rafts by PUFAs, and is thought to thereby maintain their structure and function [23, 54].

**Fig. 11:**
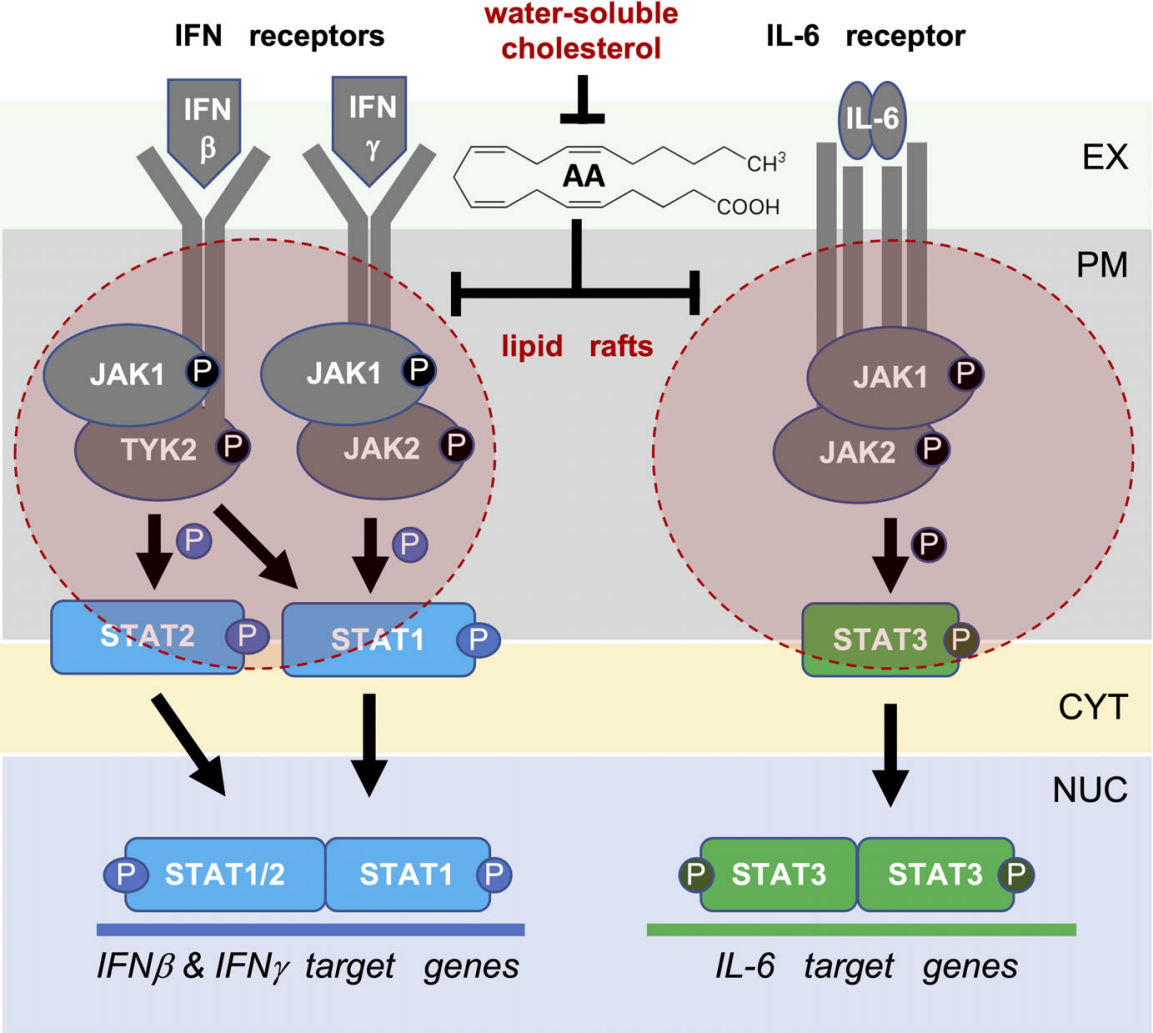
Model of arachidonic acid (AA) regulated signal transduction pathways triggered by pro‐inflammatory mediators. AA interferes with the lipid‐raft association of pro‐inflammatory cytokine receptors, receptor‐associated JAK protein kinases and STAT proteins. This mislocalization impairs the cytokine‐triggered phosphorylation and activation of JAK1/2 and STAT1/3, and thereby induction of their target genes. EX, extracellular space; PM, plasma membrane; CYT, cytosol; NUC, nucleus.

Taken together and in combination with the observed rapid effect of AA on STAT1 phosphorylation (Fig. S1), these observations suggest that AA at least partially exerts its inhibitory effect on cytokine‐triggered signal transduction and JAK‐STAT signaling in particular, by displacing membrane receptor and associated signal transduction proteins from lipid rafts (model in Fig. 11).

## 4 Discussion

This study provides strong evidence that AA, and to a lesser degree other PUFAs, at concentration found in OC ascites [7] inhibit JAK‐STAT‐mediated signal transduction, and thereby diminish the response of macrophages to IFNs and other ligands with crucial roles in immune regulation. This observation is of potentially high relevance, as the level of AA in ascites is associated with a short RFS [7], while the presence of cytotoxic T and NK cells, whose activation is dependent on IFNγ‐induced cytokines from macrophages, is linked to a favorable clinical outcome [55, 56]. These findings suggest that an inhibitory effect of AA on IFN‐dependent, immune‐stimulatory signaling events may contribute to the impairment of anti‐tumor surveillance.

### 4.1 Role of JAK‐STAT‐dependent signal transduction in anti‐tumor surveillance

The clinical relevance of IFN signaling in the context of OC has been suggested by multiple previous studies [57]. In accordance with such a connection, *IFNG* mRNA in OC tumor tissue [58], intratumoral interferon regulatory factor (IRF)‐1 [59] and genes linked to IFN signaling [34] have been associated with a favorable clinical outcome. Moreover, the addition of IFNγ in OC therapy triggered an effector immune cell response [60] and prolonged the RFS [61, 62]. In contrast, type I IFNs showed no clinical benefit [63], pointing to specific clinically relevant functions of IFNγ. This observation may result from the ability of IFNγ to induce the secretion of NK‐ and T‐cell‐stimulatory cytokines by macrophages, as tumor‐infiltrating CD8^+^ T cells are clearly linked to a long overall survival (OS) of OC [64, 65, 66], as are effector memory CD8^+^ cells and NK cells in ascites [55, 56].

IL‐12 may play a key role in this context, as it not only triggers the differentiation and activation of CD8+T cells and NK cells [67], but also appears to be linked to a favorable OC outcome, as suggested by both mouse models [68, 69] and clinical observations [70]. Expression of the IL‐12B subunit is upregulated by TLR ligands, which are also abundant in the TME [71] and may potentially contribute to macrophage activation. However, TLR‐induced signal transduction is also inhibited by AA, as shown for LPS in this study, which appears to contribute to a compromised anti‐tumor response.

IFNs and TLR ligands also induce numerous other immune stimulatory factors, in particular chemokines that mediate the local attraction of other immune cells. Among these, the CXCR4‐binding chemokines CXCR9, CXCR10, and CXCR11 are of particular interest, as they attract effector T cells to the tumor site, and, consistently, are associated with a favorable RFS of OC [55]. The AA‐mediated repression of their JAK‐STAT‐dependent induction, as shown in the present study (Figs 1, 2, and 7), may therefore represent another relevant determinant of the diminished or defective anti‐tumor immune surveillance in the OC TME.

### 4.2 Impact of AA on JAK‐STAT‐dependent signal transduction

Previous publications have reported the insertion of AA and other PUFAs into lipid rafts, leading to alterations of their lipid and protein composition, including membrane receptors and receptor‐activated protein kinases [23, 24, 72, 73, 74]. This is consistent with our own data which revealed a dramatic increase in free AA in lipid rafts after a 1‐h exposure of MDMs (Fig. 9E). Likewise, our observation that inhibition of phospholipid synthesis by Triacsin C did not affect the inhibitory effect of AA on IFN signaling (Fig. 9A–D) supports the conclusion that free AA insertion into lipid rafts is mechanistically crucial.

The relevance of lipid rafts in the context of INF signaling has been implied by the study of Sen et al. [75], who reported that Leishmania infection of macrophages causes increased membrane fluidity in conjunction with perturbed IFNγ receptor subunit assembly, which was reversible by restoration of raft structures by exogenous liposomal cholesterol. The exclusion of IFN receptor and STAT proteins from lipid rafts by AA in MDMs, as suggested by the data in Fig. 8, is in line with these previous findings. Our proteomic analysis also found STAT1, an essential transducer of IFNγ signals, to be excluded from lipid rafts upon AA treatment, which is consistent with its previous description as a caveolae‐localized protein [76].

We also identified IL‐6‐triggered signaling via STAT3 as a pathway targeted by AA, which is presumably inhibited via an analogous mechanism as discussed for IFNγ and STAT1 above. This is supported by the essential role of caveolae in IL‐6‐triggered signaling in multiple myeloma cells, as shown by its abrogation by cholesterol depletion [77], and by the localization of the IL‐6 receptor and STAT3 protein to the lipid raft compartment in a prostate cancer cell line [78].

Previous studies have shown that PUFAs displace cholesterol from lipid rafts, and that this structural perturbance can be structurally and functionally reversed by the exogenous supply of the water‐soluble Chol/MCD complex to endothelial cells and keratinocytes [23, 54], or by the liposomal delivery of cholesterol to macrophages [75]. We made use of these observations to functionally link the AA‐mediated defect in JAK‐STAT signaling to lipid rafts by clearly demonstrating rescued STAT1 phosphorylation in MDMs stimulated with IFNβ or INFγ in the presence of AA (Fig. 10). Taken together with the association of IFNγ signaling and AA with immune suppression and OS of OC, our findings are potentially relevant with respect to understanding OC progression and the development of improved therapeutic strategies.

### 4.3 Impact of AA on TLR4‐initiated signal transduction

TLR ligands represent another crucial group of pro‐inflammatory signaling molecules acting on macrophages, such as TLR4 receptors activated by LPS. TLRs signal via multiple transduction pathways, including STAT1 [79], and in agreement with this observation, our results showed a clear inhibition by AA of the LPS‐mediated phosphorylation of STAT1 and the majorly STAT1‐dependent LPS target gene *CXCL10* (Fig. 7). Previous studies have also shown that TLR4 and lipid raft proteins cooperate in LPS‐induced pro‐inflammatory signaling [80], and that TLR4 recruitment into lipid rafts is modulated by PUFAs [73]. Activation of TLR4 is preceded by binding of LPS to CD14 (and probably CD36) in lipid rafts, followed by the transfers of LPS to the TLR4 receptor complex, which dimerizes and triggers multiple transduction pathways, with NFκB and ERK playing a predominant role [72]. The association of TLR4 with lipid rafts suggests that the majority of LPS target genes, including those that are mainly regulated by NFκB and ERK, should be repressed by AA, if the hypothesis that the molecule displaces crucial LPS‐signaling signaling components from lipid rafts is valid. We were able to confirm this prediction by RNA‐Seq and phosphoprotein analyses. AA impaired the induction of most LPS target genes (Fig. S4A–C; Table S8), including *IL12B*, which is only weakly regulated via STAT1, but strongly dependent on ERK (Fig. S5). Notably, AA inhibited not only LPS‐induced *IL12B* RNA expression, but also IL‐12B secretion (Fig. S4D). Furthermore, our data revealed a clear inhibition by AA of ERK phosphorylation (Fig. S6A) and NFκB activation, the latter documented by diminished p65 (RelA) phosphorylation (Fig. S6B) and increased IκBα and IκBβ levels (Fig. S7) upon AA treatment. These results strongly confirm the view that AA interferes with the lipid‐raft localization of TLR4, thereby perturbing all TLR4‐riggered signal transduction events.

We were also interested to investigate whether signaling pathways not involving STAT proteins might be affected by AA. We focused on TGFβ due to its critical role in promoting alternative macrophage activation and thus in the reeducation of TAMs [81, 82]. As shown in Fig. S8, phosphorylation of SMAD2, a crucial step in TGFβ signal transduction, was not affected by AA, and consistently induction of the TGFβ target genes *SMAD7, ID3, OLR1,* and *RGS1* remained unchanged in the presence of AA (Fig. S9). These observations suggest that AA interferes predominantly with pro‐infammatory signaling in macrophages and thereby contributes to the immunosuppressed phenotype of TAMs, and thus to an inhibition of cytotoxic immune response by T and NK cells, for instance, by blocking IL‐12 secretion.

## 5 Conclusions

Our data suggest that AA impairs pro‐inflammatory signal transduction in macrophages triggered by diverse mediators, including IFNs, IL‐6 and TLR ligands. The inhibitory effect of AA on IFN signaling by impairing the receptor‐JAK‐STAT axis is likely to be particularly relevant in the context of the OC TME, as it may contribute to the immunosuppressive reeducation of TAMs. As an underlying mechanism, we propose the AA‐mediated alteration of the composition of lipid rafts, including the exclusion of signaling molecules transducing cytokine and TLR signals. As IFNγ signaling and AA levels in the TME are linked to OC progression, our findings provide the basis for novel therapeutic approaches. These may, for example, involve the pharmacologic restoration of lipid raft functions in TAMs in combination with strategies targeting other mediators in the TME inhibiting TAM functions.

## Conflict of interest

The authors declare no conflict of interest.

## Author contributions

RM, SR, and SM‐B designed the study and supervised the project. MKH and RD performed immunoblotting experiments. AU and TB performed initial experiments providing the basis for the present study. JG performed MS‐based phosphoproteomics. AN and TS carried out RNA‐Sequencing. RD, MKH, FF, AMB, JG, and RM analyzed the raw data. MKH, FF, and RM carried out bioinformatic analyses. RM wrote the manuscript.

## Supporting information


**Fig. S1.** Persistence of AA‐mediated inhibition of IFNγ‐induced STAT1 phosphorylation.
**Fig. S2.** Concentration dependence of the AA‐mediated inhibition of cytokine‐induced STAT phosphorylation.
**Fig. S3.** Lipidomic analysis of lipid rafts.
**Fig. S4.** Impact of AA on the transcriptome of LPS‐stimulated MDMs.
**Fig. S5.** Repression of JAK/STAT‐independent LPS target gene *IL12B* by AA.
**Fig. S6.** Inhibition of LPS‐induced ERK and NFκB signaling in MDMs by AA and ETYA.
**Fig. S7.** Inhibition of LPS‐induced degradation of IκBα and IκBβ in MDMs by AA and ETYA.
**Fig. S8.** TGFβ‐induced SMAD2 phosphorylation is not affected by AA.
**Fig. S9.** Impact of AA on the transcriptome of TGFβ‐stimulated MDMs.

**Table S1.** qRT‐PCR primers.
**Table S2.** RNA‐Seq data for genes inversely correlated with CD163 / CD206 (MRC1).
**Table S3.** RNA‐Seq data for genes genes induced by INFβ after preincubation with AA or solvent.
**Table S4.** RNA‐Seq data for genes genes induced by INFγ after preincubation with AA or solvent.
**Table S5.** RNA‐Seq data for genes genes induced by IL‐6 after preincubation with AA or solvent.
**Table S6.** Proteomic analysis of lipid rafts from MDMs treated with AA or ETYA versus solvent.
**Table S7.** Reactome pathway analysis of proteins missing in lipid rafts from AA/ETYA‐treated MDMs.
**Table S8.** RNA‐Seq data for genes genes induced by LPS after preincubation with AA or solvent.

## Data Availability

RNA‐Seq data were deposited at EBI ArrayExpress (accession numbers E‐MTAB‐4162, E‐MTAB‐5498). Proteomic data have been deposited at the PRIDE partner repository (dataset identifier PXD028434).
