# Supplementary material for: Arachidonic acid, a clinically adverse mediator in the ovarian cancer microenvironment, impairs JAK‐STAT signaling in macrophages by perturbing lipid raft structures
Source: Mol Oncol. 2022 May 4;16(17):3146–66. doi: 10.1002/1878-0261.13221 (PMC9441005; doi:10.1002/1878-0261.13221)
Supplement: Supplementary file 1 — Fig. S1. Persistence of AA‐mediated inhibition of IFNγ‐induced STAT1 phosphorylation. Fig. S2. Concentration dependence of the AA‐mediated inhibition of cytokine‐induced STAT phosphorylation. Fig. S3. Lipidomic analysis of lipid rafts. Fig. S4. Impact of AA on the transcriptome of LPS‐stimulated MDMs. Fig. S5. Repression of JAK/STAT‐independent LPS target gene IL12B by AA. Fig. S6. Inhibition of LPS‐induced ERK and NFκB signaling in MDMs by AA and ETYA. Fig. S7. Inhibition of LPS‐induced degradation of IκBα and IκBβ in MDMs by AA and ETYA. Fig. S8. TGFβ‐induced SMAD2 phosphorylation is not affected by AA. Fig. S9. Impact of AA on the transcriptome of TGFβ‐stimulated MDMs. [file MOL2-16-3146-s001.pdf]

SUPPLEMENTAL FIGURES

Arachidonic acid, a clinically adverse mediator in the ovarian cancer microenvironment, impairs JAK-STAT signaling in macrophages by perturbing lipid raft structures

Mohamad K. Hammoud, Raimund Dietze, Jelena Pesek, Florian Finkernagel, Annika Unger, Tim Bieringer, Andrea Nist, Aditya M. Bhagwat, Thorsten Stiewe, W. Andreas Nockher, Silke Reinartz, Sabine Müller-Brüsselbach, Johannes Graumann and Rolf Müller

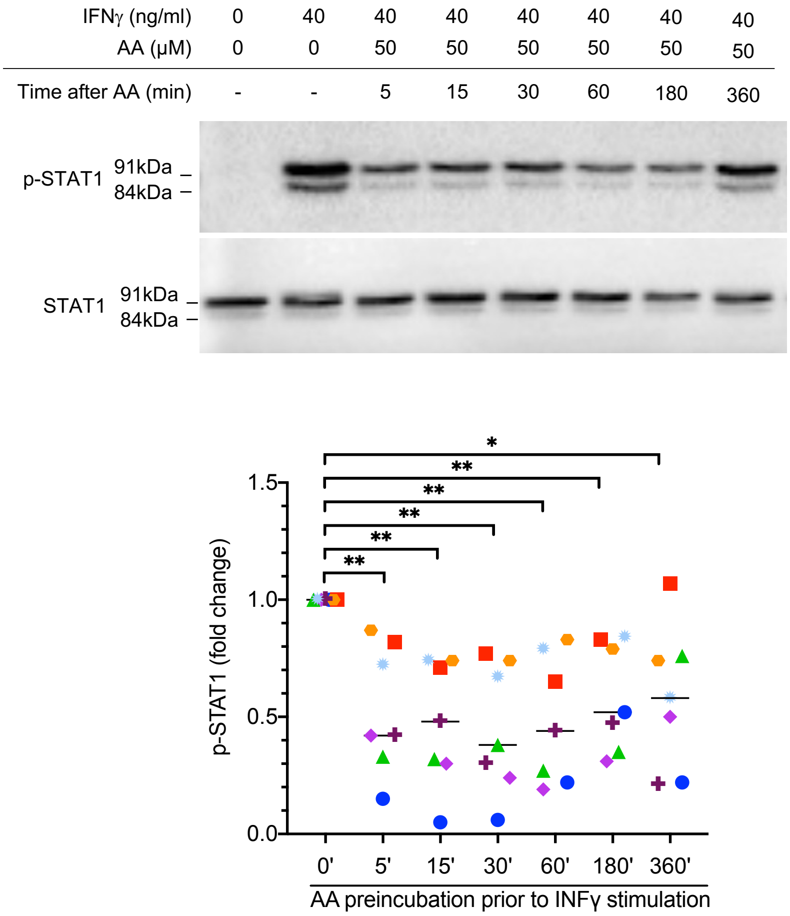

Figure S1: Persistence of AA-mediated inhibition of IFNγ-induced STAT1 phosphorylation.

Immunoblot analysis of the IFNγ-induced phosphorylation of STAT1 (Y701) for 30 min after preincubation with 50 µM AA for different time points. A representative immunoblot and the quantification of n=7 independent experiments (different donors; represented by different symbols) are shown. Statistical significance was analyzed by paired t test (*$p<0.05$; **$p<0.01$).

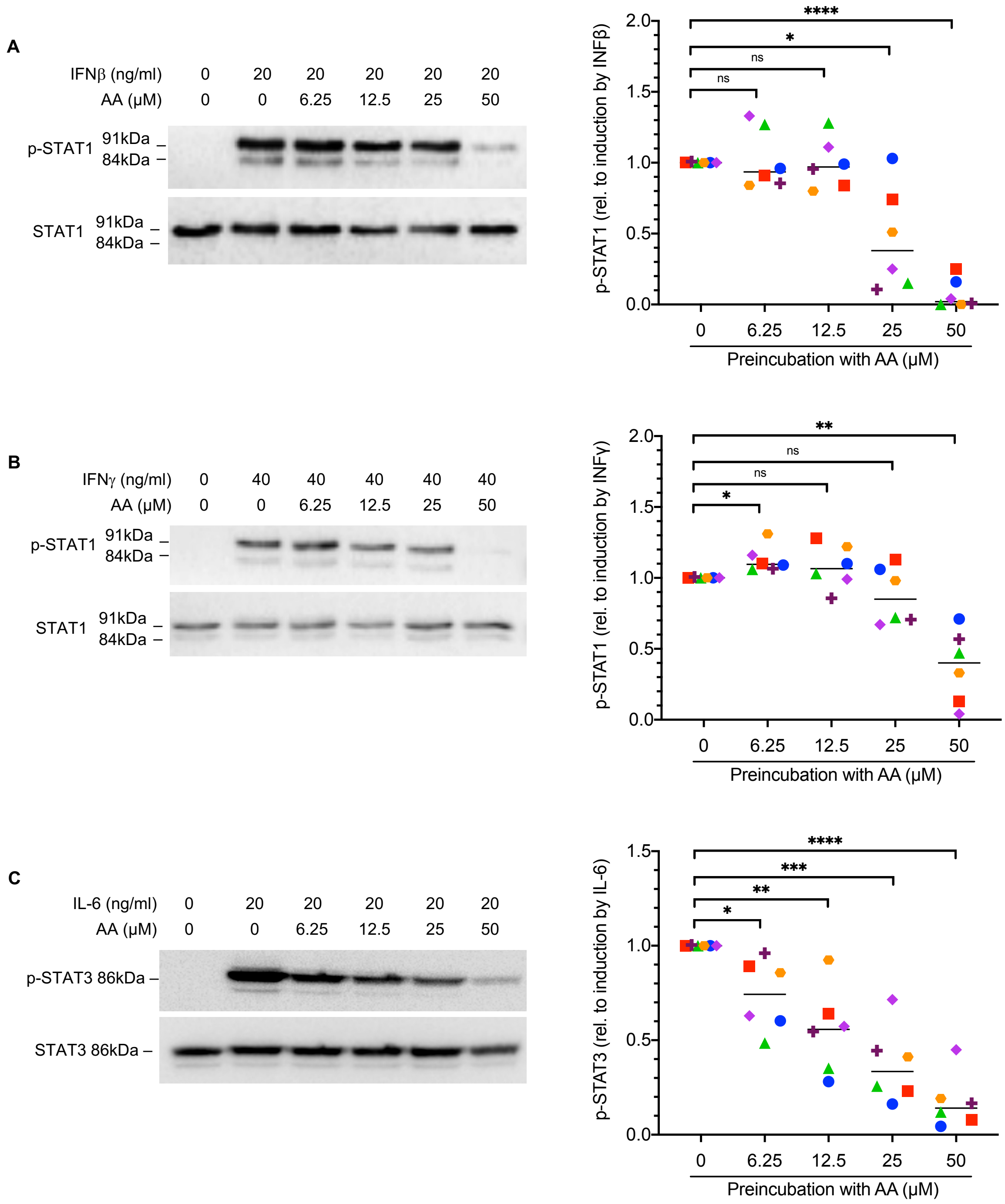

Figure S2: Concentration dependence of the AA-mediated inhibition of cytokine-induced STAT phosphorylation.

(A, B) Immunoblot analysis of IFNβ-induced and IFNγ-induced phosphorylation of STAT1 (Y701) following preincubation with AA. **(C)** Immunoblot analysis of IL-6-induced phosphorylation of STAT3 (Y705) following preincubation with AA. In each experimental setup, MDMs were pretreated with the indicated concentration of AA for 30 min prior to stimulation with the IFNβ, IFNγ or IL-6 for 30 min. Representative immunoblots and the quantification of n=6 independent experiments (different donors; represented by different symbols) are shown in each panel. Statistical significance was analyzed by paired t test (*p<0.05; **p<0.01; ***p<0.001; ****p<0.0001; ns, not significant).

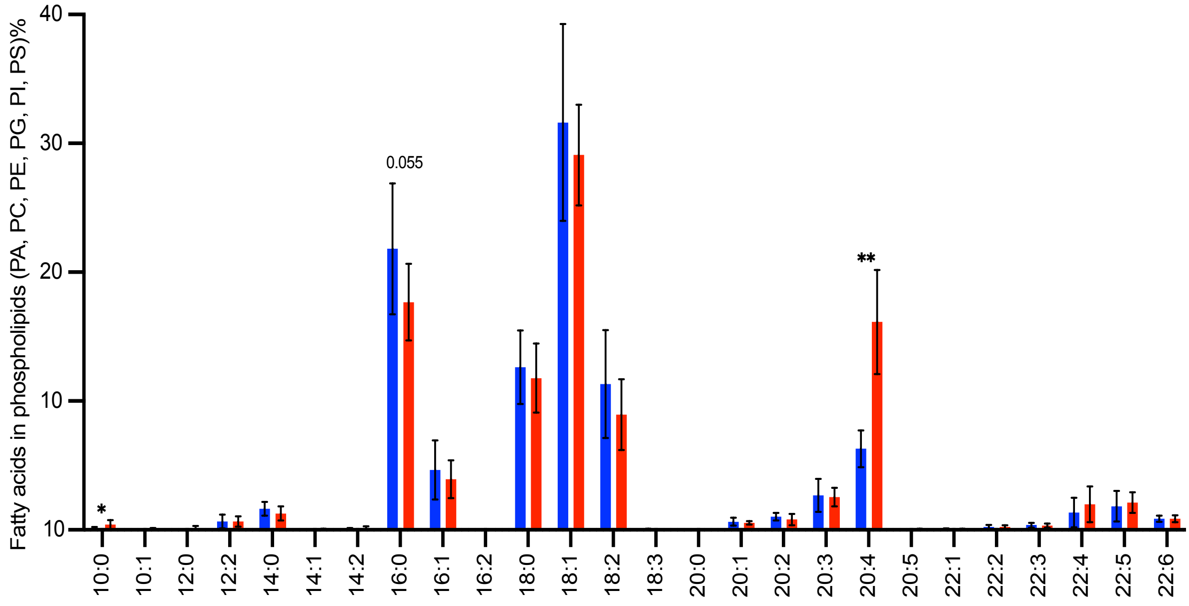

Figure S3: Lipidomic analysis of lipid rafts.

Analysis of phospholipids from lipid rafts of MDMs after treatment with 50 µM AA or solvent for 1 hr. The plot shows the percentage of specific fatty acids differing in length and saturation (indicated below x-axis) in phospholipids atty acids differing in length and saturation (indicated below x-axis) in phospholipids (combined; PA: phosphatidic acid, PC: phosphatidylcholine, PE: phosphatidylethanolamine, PG: phosphatidylglycerol, PI: phosphatidylinositol and PS phosphatidylserine). Statistical significance was analyzed by paired t test (*p<0.05; **p<0.01; ns: not significant; n=6).

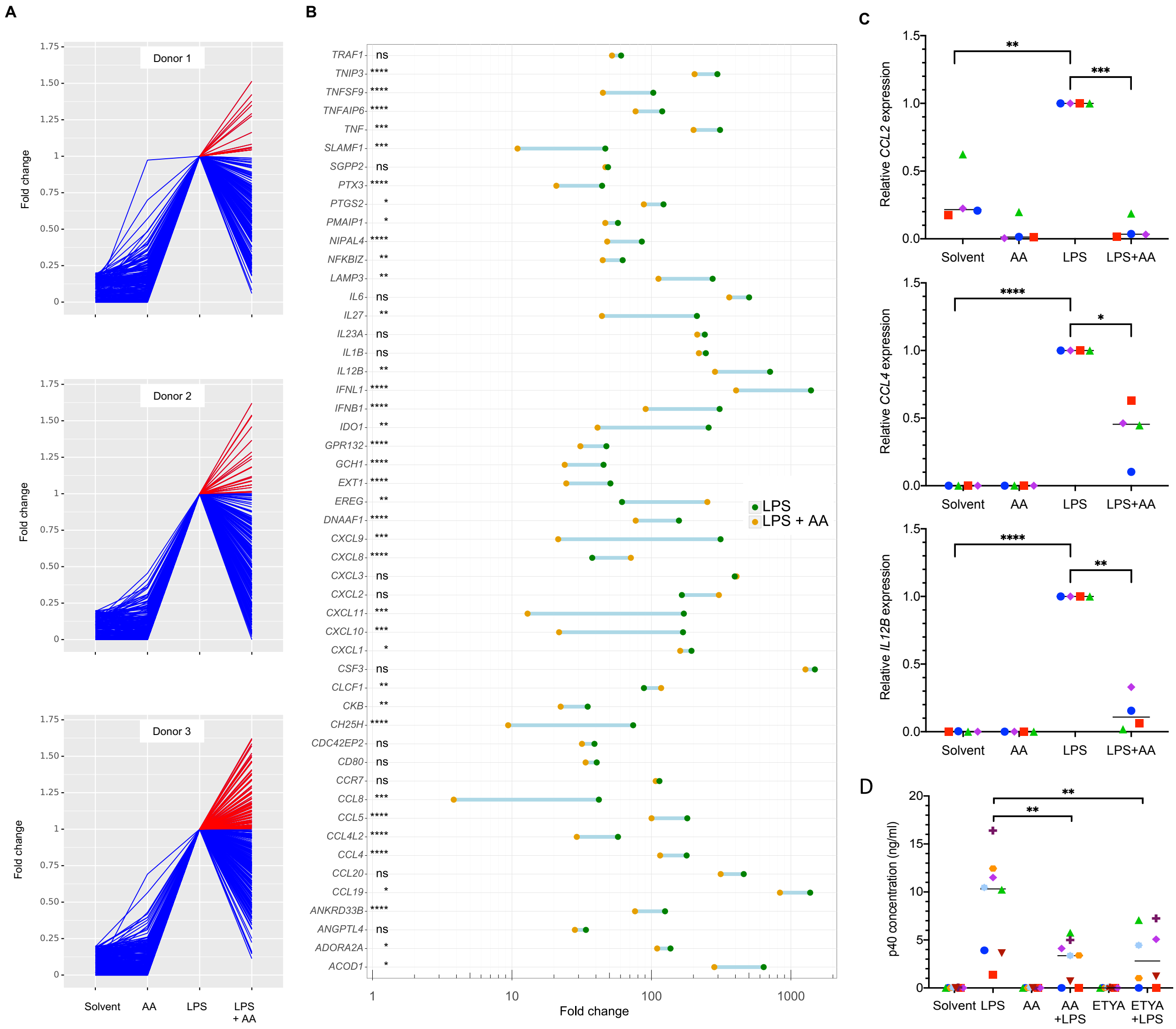

Figure S4: Impact of AA on the transcriptome of LPS-stimulated MDMs.

MDMs were pretreated with 50 µM AA or solvent for 30 min prior to stimulation with 100 ng/ml LPS for 3 hrs followed by RNA-Seq analysis. **(A)** RNA-Seq results for the top LPS-induced genes (FC ≥5 for LPS versus solvent; CPM ≥5 for LPS-stimulated cells). Data were normalized for LPS-stimulated cells, and data points were connected by lines for improved visualization. Blue: LPS-induced genes repressed by AA; red: LPS-induced genes upregulated by AA. The three plots depict the data for n=3 donors. **(B)** LPS-induced genes showing the strongest repression by AA (top 50 LPS induced genes and FDR ≤0.05 for LPS versus LPS plus AA). The green and orange data points show the mean (n=3) induction values for LPS and LPS plus AA, respectively. **(C)** Validation of RNA-Seq results by qRT-PCR for *CCL2, CCL4* and *IL12B* using *RPL27* as the normalizer. Cy0 values are expressed relative to LPS-stimulated cells for n=4 donors (represented by different symbols). **(D)** Repression of the LPS-induced secretion of IL-12B/p40 by AA and ETYA. MDMs from n=7 donors were incubated with LPS (100ng/ml) for 24 hrs after preincubation with 50 µM of AA or ETYA for 30 min, and culture supernatants were analyzed by ELISA. Statistical significance was analyzed by paired t test (*p<0.05; **p<0.01; ***p<0.001; ****p<0.0001).

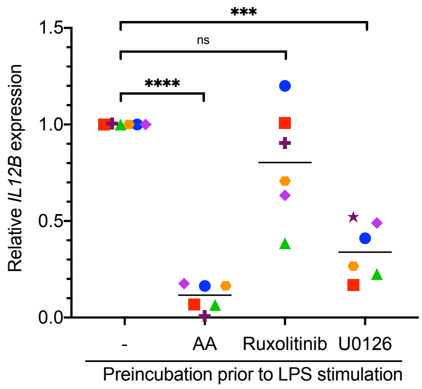

Figure S5: Repression of JAK/STAT-independent LPS target gene *IL12B* by AA.

MDMs were pretreated with 50 µM AA, the JAK inhibitor Ruxolitinib (0.5 µM) or the ERK inhibitor UO126 (10 µM) for 30 min prior to stimulation with 100 ng/ml LPS for 3 hrs and analyzed by RT-qPCR. Cy0 values are expressed relative to LPS-stimulated cells for n=6 donors (represented by different symbols). Statistical significance was analyzed by paired t test (***p<0.001; ****p<0.0001; ns: not significant).

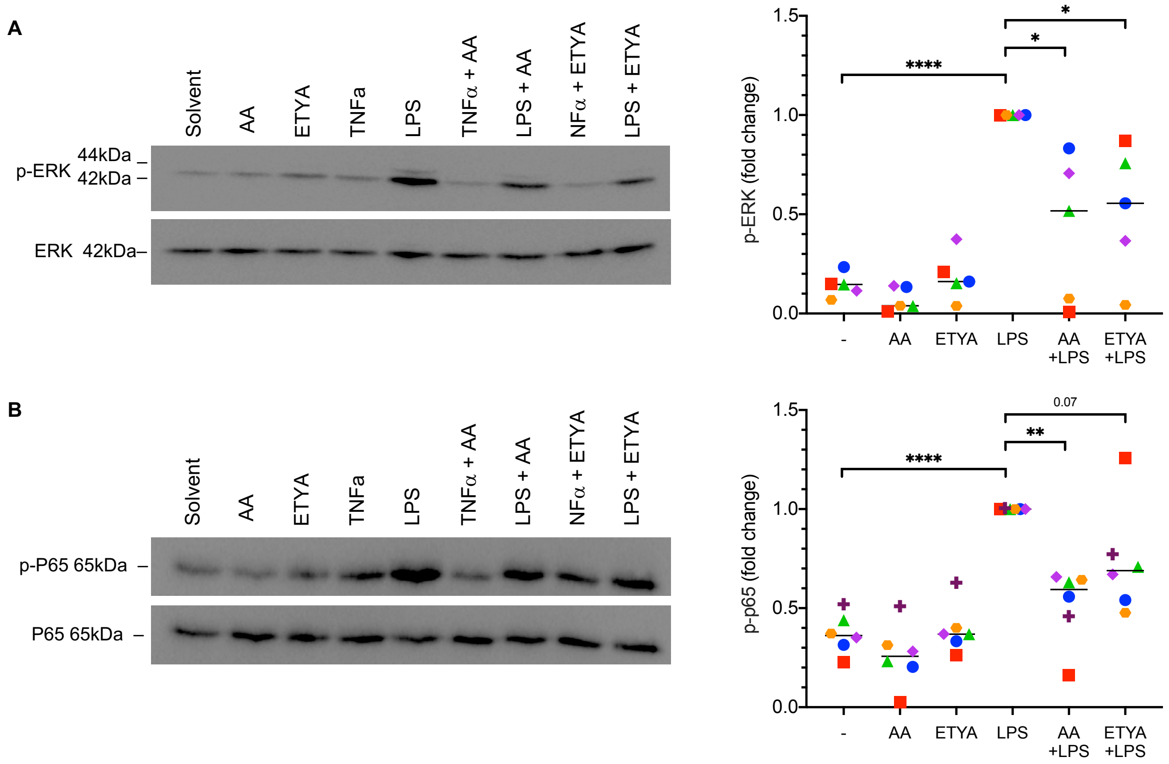

Figure S6: Inhibition of LPS-induced ERK and NFκB signaling in MDMs by AA and ETYA.

(A) Inhibition of LPS-induced phosphorylation of ERK1/2 (Thr202/Tyr204). Only one ERK band is visible most likely due the low expression of ERK1 (~15% of ERK2 according to our proteomics data; see www.ovara.net). **(B)** Inhibition of LPS-induced phosphorylation of p65 by AA (Ser536). MDMs were pretreated with 50 µM of AA, ETYA or solvent for 30 min prior to stimulation with 100 ng/ml LPS for 30 min. Representative immunoblots and quantifications of n=5 (A) and n=6 (B) replicates, respectively, are shown (individual donors represented by different symbols). Statistical significance was analyzed by paired t test (*p<0.05; **p<0.01; ****p<0.0001).

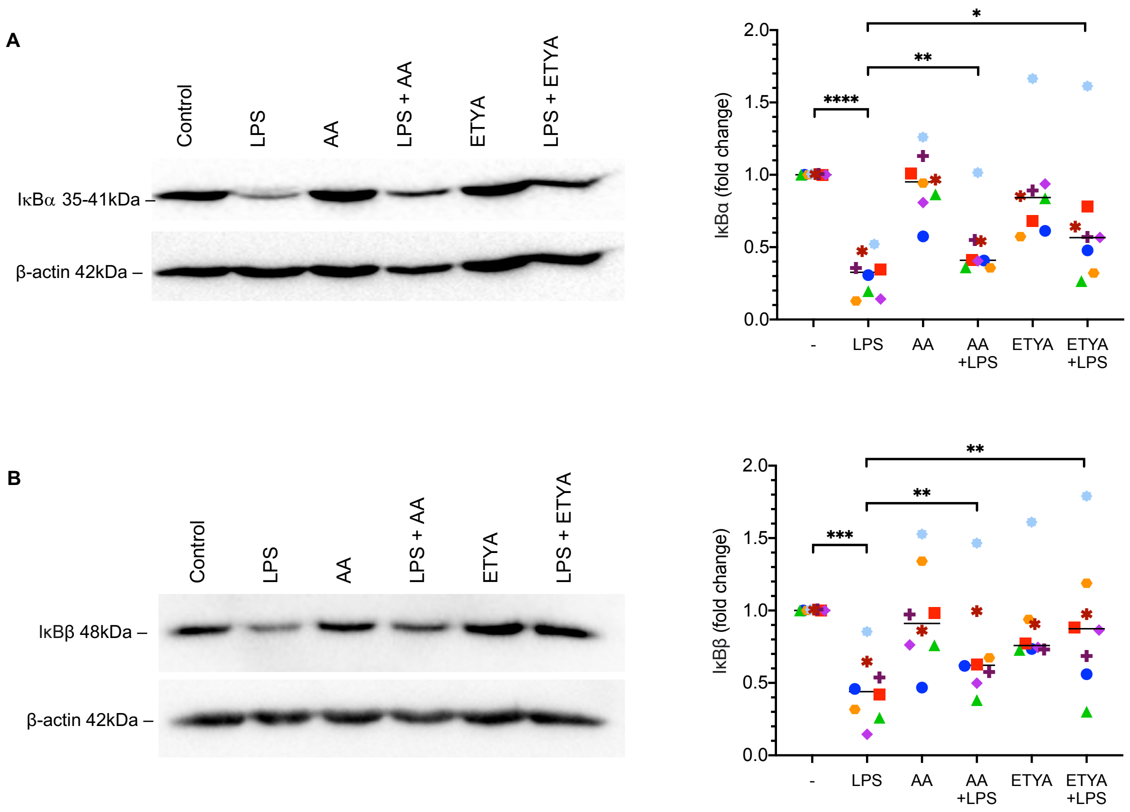

Figure S7: Inhibition of LPS-induced degradation of IκBα and IκBβ in MDMs by AA and ETYA.

(A) Inhibition of LPS-induced degradation of IκBα. **(B)** Inhibition of LPS-induced degradation of IκBβ. MDMs were pretreated with 50 µM of AA, ETYA or solvent for 30 min prior to stimulation with 100 ng/ml LPS for 60 min. Representative immunoblots and quantifications of n=8 replicates are shown (individual donors represented by different symbols). Statistical significance was analyzed by paired t test (*$p<0.05$; **$p<0.01$; ***$p<0.001$; ****$p<0.0001$).

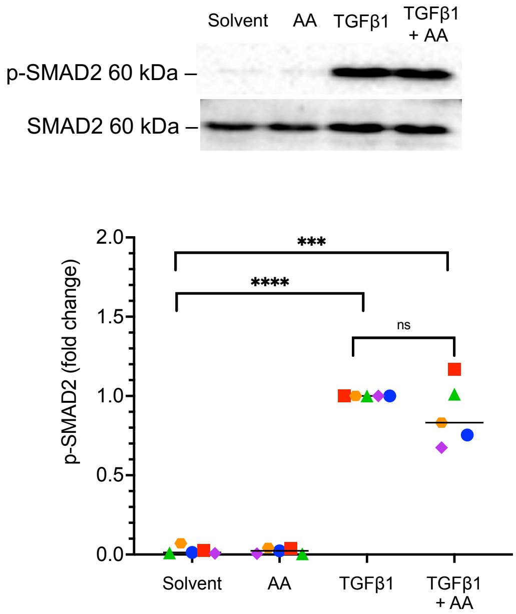

Figure S8: TGFβ-induced SMAD2 phosphorylation is not affected by AA.

MDMs were pretreated with 50 µM of AA or solvent for 30 min prior to stimulation with 35 ng/ml TGFβ for 30 min. Representative immunoblots and quantifications of n=5 replicates are shown (individual donors represented by different symbols). Statistical significance was analyzed by paired t test (***$p<0.001$; ****$p<0.0001$).

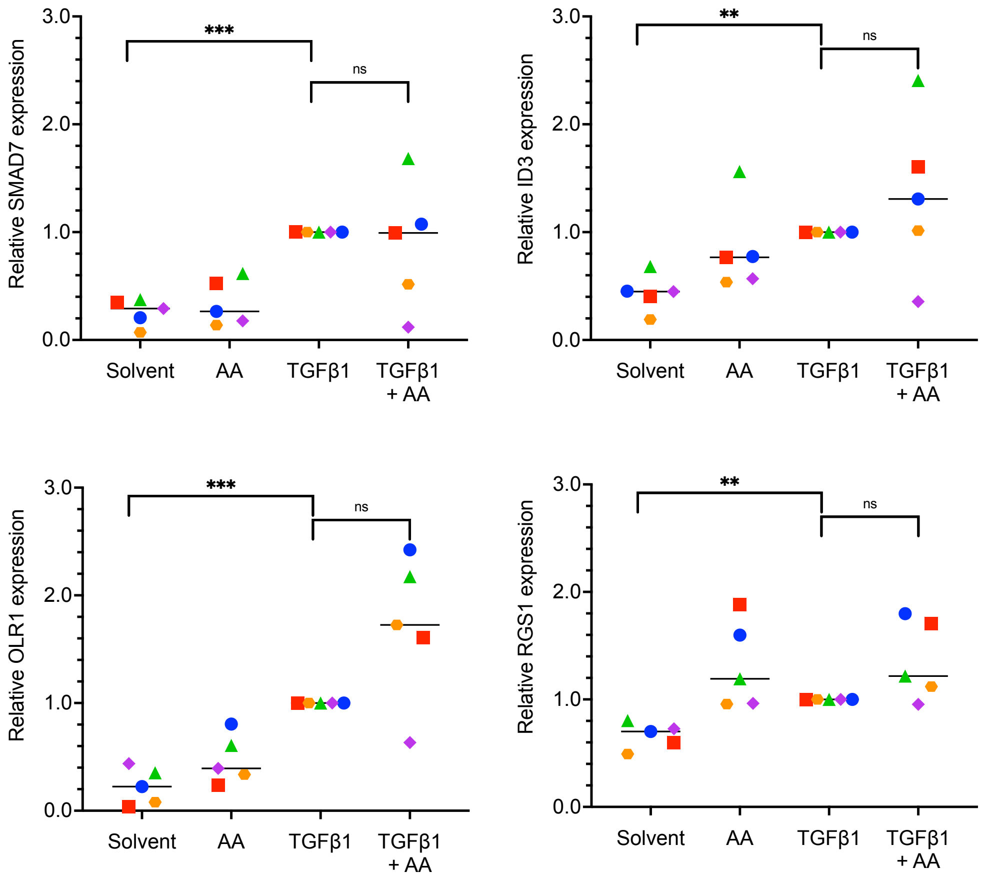

Figure S9: Impact of AA on the transcriptome of TGFβ-stimulated MDMs.

MDMs were pretreated with 50 µM of AA or solvent for 30 min prior to stimulation with 35 ng/ml TGFβ for 3 hrs followed by qRT-PCR analysis of the TGFβ target genes *SMAD7, ID3, OLR1* and *RGS1* using *RPL27* as the normalizer. Cy0 values are expressed relative to TGFβ-stimulated cells for n=5 donors (represented by different symbols). Statistical significance was analyzed by paired t test (**p<0.01; ***p<0.001; ns: not significant).
