# Supplementary material for: Arachidonic acid, a clinically adverse mediator in the ovarian cancer microenvironment, impairs JAK‐STAT signaling in macrophages by perturbing lipid raft structures
Source: Mol Oncol. 2022 May 4;16(17):3146–66. doi: 10.1002/1878-0261.13221 (PMC9441005; doi:10.1002/1878-0261.13221)
Supplement: Supplementary file 3 [file MOL2-16-3146-s003.pdf]

**LC-MSMS Parametrization**
This file is produced using R and the package openxlsx .

**Tab Index**

| | |
|---|---|
| LC-MSMS Parameters: | LC-MS/MS parameters extracted from ThemeroFisher Scientific RAW files using |
| LC Gradient: | Chromatography gradient(s) extracted from ThermoFisher Scientific RAW files using (Kiweler, |
| MaxQuant Parameters: | Parametrization of the MaxQuant suite of algorithms (Cox and Mann, 2008; Cox, Hein, Luber, Paron, Nagaraj, and ann, Matthias, 2014; |

**Bibliography**

[1] J. Cox, M. Y. Hein, C. A. Luber, et al. "Accurate Proteome-wide Label-free Quantification by Delayed Normalization and Maximal Peptide Ratio Extraction, Termed MaxLFQ". In: _Molecular & Cellular Proteomics_ 13.9 (2014), pp. 2513-2526. DOI: 10.1074/mcp.M113.031591.

[2] J. Cox and M. Mann. "MaxQuant enables high peptide identification rates, individualized p.p.b.-range mass accuracies and proteome-wide protein quantification". In: _Nature Biotechnology_ 26.12 (2008), pp. 1367-1372. DOI: 10.1038/nbt.1511.

[3] J. Cox, N. Neuhauser, A. Michalski, et al. "Andromeda: A Peptide Search Engine Integrated into the MaxQuant Environment". In: _Journal of Proteome Research_ 10.4 (2011), pp. 1794-1805. DOI: 10.1021/pr101065j.

[4] M. Kiweler, M. Looso, and J. Graumann. "MARMoSET – Extracting Publication-Ready Mass Spectrometry Metadata from RAW Files". In: _Molecular & Cellular Proteomics_ (Jan. 01, 2019), p. mcp.TIR119.001505. DOI: 10.1074/mcp.TIR119.001505.

| Term | Value |
|---|---|
| High Performance Liquid Chromatography (HPLC) Instrument | Thermo EASY-nLC |
| HPLC Vendor | Thermo Scientific |
| Injected Sample Volume | 4.00 |
| Mass Spectrometer (MS) | Q Exactive HF - Orbitrap MS |
| MS Vendor | Thermo Scientific |
| MS Model | QExactive HF |
| MS Instrument Software | Xcalibur |
| MS Instrument Software Version | 2.11-211200/2.11.0.3007 |
| MS Ionization Type | Electrospray Ionization (ESI) |
| MS Electrospray Voltage | 1900 |
| MS Heated Capillary Temperature | 350 |
| MS Analyzer Precursor Ion Spectrum (MS1) | orbitrap |
| MS Data Type Precursor Ion Spectrum (MS1) | Profile |
| MS Micro Spectra Precursor Ion Spectrum (MS1) | 1 |
| MS Resolution Precursor Ion Spectrum (MS1) | 60,000 |
| MS Resolution m/z Precursor Ion Spectrum (MS1) | 200 m/z |
| MS Mass Range Precursor Ion Spectrum (MS1) | 300 to 1650 m/z |
| MS Ion Target Value Precursor Ion Spectrum (MS1) | 3e6 |
| MS Max. Injection Time Precursor Ion Spectrum (MS1) | 15 ms |
| MS Analyzer Fragment Ion Spectrum (MS2) | orbitrap |
| MS Data Type Fragment Ion Spectrum (MS2) | Profile |
| MS Micro Spectra Fragment Ion Spectrum (MS2) | 1 |
| MS Fixed First m/z Fragment Ion Spectrum (MS2) | - |
| MS Isolation Window Width Fragment Ion Spectrum (MS2) | 1.2 m/z |
| MS Isolation Window Offset Fragment Ion Spectrum (MS2) | 0.0 m/z |
| MS Fragmentation Mode (MS2) | HCD |
| MS Normalized Collision Energy Fragment Ion Spectrum (MS2) | nce: 28 |
| MS Resolution Fragment Ion Spectrum (MS2) | 15,000 |
| MS Resolution m/z Fragment Ion Spectrum (MS2) | 200 m/z |
| MS Ion Target Value Fragment Ion Spectrum (MS2) | 5e5 |
| MS Max. Injection Time Fragment Ion Spectrum (MS2) | 15 ms |
| MS Loop Count (Top n) Fragment Ion Spectrum (MS2) | 20 |
| MS Charge States Excluded from Fragmentation | unassigned, 1, >8 |
| MS Minimum Peak Intensity for Fragmentation | 1.0e5 |
| MS Fragmentation Trigger on Peak Apex | - |
| MS Dynamic Exclusion Length | 20.0 s |
| MS Isotopologue Exclusion | on |
| MS Peptide Model for Excluded Isotope Clusters | preferred |

| Time[mm:ss] | Duration[mm:ss] | Flow[nl/min] | Mixture[%B] |
|---|---|---|---|
| 00:00 | 00:00 | 300 | 5 |
| 05:00 | 05:00 | 300 | 5 |
| 125:00 | 120:00 | 300 | 38 |
| 132:00 | 07:00 | 300 | 60 |
| 138:00 | 06:00 | 300 | 95 |
| 143:00 | 05:00 | 300 | 95 |
| 148:00 | 05:00 | 300 | 5 |
| 150:00 | 02:00 | 300 | 5 |

| Parameter | Value |
|---|---|
| Version | 1.6.17.0 |
| User name | aklaus |
| Machine name | KI-S0114 |
| Date of writing | 07/10/2021 20:03:29 |
| Include contaminants | True |
| PSM FDR | 0.01 |
| PSM FDR Crosslink | 0.01 |
| Protein FDR | 0.01 |
| Site FDR | 0.01 |
| Use Normalized Ratios For Occupancy | True |
| Min. peptide Length | 7 |
| Min. score for unmodified peptides | 0 |
| Min. score for modified peptides | 40 |
| Min. delta score for unmodified peptides | 0 |
| Min. delta score for modified peptides | 6 |
| Min. unique peptides | 0 |
| Min. razor peptides | 1 |
| Min. peptides | 1 |
| Use only unmodified peptides and | True |
| Modifications included in protein quantification | Oxidation (M);Acetyl (Protein N-term) |
| Peptides used for protein quantification | Razor |
| Discard unmodified counterpart peptides | True |
| Label min. ratio count | 2 |
| Use delta score | False |
| iBAQ | False |
| iBAQ log fit | False |
| Match between runs | True |
| Matching time window [min] | 0.7 |
| Match ion mobility window [indices] | 0.05 |
| Alignment time window [min] | 20 |
| Alignment ion mobility window [indices] | 1 |
| Find dependent peptides | False |
| Fasta file | \\ki-s0211.kerckhoff.mpg.de\AG-Proteomics$\Fasta 2021\HUMAN_9606_can+iso_20210208_194237.fasta |
| Decoy mode | revert |
| Include contaminants | True |
| Advanced ratios | True |
| Fixed andromeda index folder | |
| Combined folder location | |
| Second peptides | False |
| Stabilize large LFQ ratios | True |
| Separate LFQ in parameter groups | False |
| Require MS/MS for LFQ comparisons | True |
| Calculate peak properties | False |
| Main search max. combinations | 200 |

| | |
|---|---|
| Advanced site intensities | True |
| Write msScans table | False |
| Write msmsScans table | True |
| Write ms3Scans table | True |
| Write allPeptides table | True |
| Write mzRange table | True |
| Write DIA fragments table | False |
| Write pasefMsmsScans table | True |
| Write accumulatedPasefMsmsScans table | False |
| Max. peptide mass [Da] | 4600 |
| Min. peptide length for unspecific search | 8 |
| Max. peptide length for unspecific search | 25 |
| Razor protein FDR | True |
| Disable MD5 | False |
| Max mods in site table | 3 |
| Match unidentified features | False |
| Epsilon score for mutations | |
| Evaluate variant peptides separately | True |
| Variation mode | None |
| MS/MS tol. (FTMS) | 20 ppm |
| Top MS/MS peaks per Da interval. (FTMS) | 12 |
| Da interval. (FTMS) | 100 |
| MS/MS deisotoping (FTMS) | True |
| MS/MS deisotoping tolerance (FTMS) | 7 |
| MS/MS deisotoping tolerance unit (FTMS) | ppm |
| MS/MS higher charges (FTMS) | True |
| MS/MS water loss (FTMS) | True |
| MS/MS ammonia loss (FTMS) | True |
| MS/MS dependent losses (FTMS) | True |
| MS/MS recalibration (FTMS) | False |
| MS/MS tol. (ITMS) | 0.5 Da |
| Top MS/MS peaks per Da interval. (ITMS) | 8 |
| Da interval. (ITMS) | 100 |
| MS/MS deisotoping (ITMS) | False |
| MS/MS deisotoping tolerance (ITMS) | 0.15 |
| MS/MS deisotoping tolerance unit (ITMS) | Da |
| MS/MS higher charges (ITMS) | True |
| MS/MS water loss (ITMS) | True |
| MS/MS ammonia loss (ITMS) | True |
| MS/MS dependent losses (ITMS) | True |
| MS/MS recalibration (ITMS) | False |
| MS/MS tol. (TOF) | 40 ppm |
| Top MS/MS peaks per Da interval. (TOF) | 10 |
| Da interval. (TOF) | 100 |
| MS/MS deisotoping (TOF) | True |
| MS/MS deisotoping tolerance (TOF) | 0.01 |
| MS/MS deisotoping tolerance unit (TOF) | Da |
| MS/MS higher charges (TOF) | True |

| | |
|---|---|
| MS/MS water loss (TOF) | True |
| MS/MS ammonia loss (TOF) | True |
| MS/MS dependent losses (TOF) | True |
| MS/MS recalibration (TOF) | False |
| MS/MS tol. (Unknown) | 20 ppm |
| Top MS/MS peaks per Da interval. (Unknown) | 12 |
| Da interval. (Unknown) | 100 |
| MS/MS deisotoping (Unknown) | True |
| MS/MS deisotoping tolerance (Unknown) | 7 |
| MS/MS deisotoping tolerance unit (Unknown) | ppm |
| MS/MS higher charges (Unknown) | True |
| MS/MS water loss (Unknown) | True |
| MS/MS ammonia loss (Unknown) | True |
| MS/MS dependent losses (Unknown) | True |
| MS/MS recalibration (Unknown) | False |
| Site tables | Oxidation (M)Sites.txt |
